# Peptide hormones in plants

**DOI:** 10.1186/s43897-024-00134-y

**Published:** 2025-01-23

**Authors:** Zhenbiao Zhang, Huibin Han, Junxiang Zhao, Zhiwen Liu, Lei Deng, Liuji Wu, Junpeng Niu, Yongfeng Guo, Guodong Wang, Xiaoping Gou, Chao Li, Chuanyou Li, Chun-Ming Liu

**Affiliations:** 1 Tobacco Research Institute, Chinese Academy of Agricultural Sciences, Qingdao, 266101 China; 2 College of Bioscience and Bioengineering, Jiangxi Agricultural University, Nanchang, 330045 China; 3 Ministry of Education Key Laboratory of Cell Activities and Stress Adaptations, Key Laboratory of Gene Editing for Breeding, School of Life Sciences, Lanzhou University, Lanzhou, 730000 China; 4 School of Life Sciences, East China Normal University, Shanghai, 200241 China; 5 College of Life Sciences, Shandong Agricultural University, Tai’an, 271018 China; 6 National Key Laboratory of Wheat and Maize Crop Science, College of Agronomy, Henan Agricultural University, Zhengzhou, 450046 China; 7 College of Life Sciences, Key Laboratory of Medicinal Resources and Natural Pharmaceutical Chemistry of Ministry of Education, Engineering Research Center of High Value Utilization of Western China Fruit Resources of Ministry of Education, Shaanxi Normal University, Xi’an, 710119 China; 8 Institute of Botany, Chinese Academy of Sciences, Beijing, 100093 China

**Keywords:** Peptide hormones, Secretions, Proteolytic processing, Post-translational modifications, Receptor-like kinase, Signal transductions

## Abstract

Peptide hormones are defined as small secreted polypeptide-based intercellular communication signal molecules. Such peptide hormones are encoded by nuclear genes, and often go through proteolytic processing of preproproteins and post-translational modifications. Most peptide hormones are secreted out of the cell to interact with membrane-associated receptors in neighboring cells, and subsequently activate signal transductions, leading to changes in gene expression and cellular responses. Since the discovery of the first plant peptide hormone, systemin, in tomato in 1991, putative peptide hormones have continuously been identified in different plant species, showing their importance in both short- and long-range signal transductions. The roles of peptide hormones are implicated in, but not limited to, processes such as self-incompatibility, pollination, fertilization, embryogenesis, endosperm development, stem cell regulation, plant architecture, tissue differentiation, organogenesis, dehiscence, senescence, plant-pathogen and plant-insect interactions, and stress responses. This article, collectively written by researchers in this field, aims to provide a general overview for the discoveries, functions, chemical natures, transcriptional regulations, and post-translational modifications of peptide hormones in plants. We also updated recent discoveries in receptor kinases underlying the peptide hormone sensing and down-stream signal pathways. Future prospective and challenges will also be discussed at the end of the article.

## Introduction

The discoveries of peptide hormones in human and animals are one of the greatest inventions in the last century. Many of these peptides such as insulin have been used extensively in pharmacy for saving people’s life and improving life quality. For a long time, peptide hormones have not been considered to be present and important in plants. Most plant hormones identified thus far, such as auxin, cytokinins, gibberellins, abscisic acid and ethylene, are low-molecule weight metabolites, with high sensitivities and critical functions at different stages of plant growth and development. However, since the first peptide hormone, systemin, was discovered in tomato by Ryan’s group in 1991 (Pearce et al. 1991), putative peptide hormones have continuously been identified in different plant species, showing their importance in both short- and long-range signal transductions. Their roles are implicated in processes such as pollination, self-incompatibility, fertilization, embryo and endosperm development, stem cell homeostasis, plant architecture, cell differentiation, organogenesis, dehiscence, senescence, plant-pathogen and plant-insect interactions, and stress responses. This article aims to provide a general overview for the discoveries, functions, chemical natures, transcriptional regulations, and post-translational modifications (PTMs) of plant peptide hormones. Attentions are also given to peptide-sensing receptor kinases and downstream signal transduction pathways.

## Identifications and classifications of plant peptide hormones

### Identifications of peptide hormones in plants

Peptide hormones are defined as small secreted polypeptide-based intercellular communication signal molecules (Lease and Walker 2006). The lengths of peptide hormones range from a few amino acids to up to 250 amino acids. Traditionally, the identifications of potential peptide hormones relied on various analytical methods such as chemical separations and bioassays, phenotype-based genetic analyses, and chromatographic detections (Cammue et al. 1992; Fletcher et al. 1999; Butenko et al. 2003). However, since peptide hormones are present with extremely low concentrations in plants, posing substantial challenges for liquid-phase separations, purifications, and biochemical analyses (Hou et al. 2014). To address these challenges, high-throughput omics approaches such as transcriptomics, proteomics and peptideomics, combined with detection methods including high-throughput mass spectrometry (MS) and in vitro assays, have been used to identify peptide hormones in plants (Yan et al. 2022). In addition, advances in bioinformatics tools and machine-learning approaches have enabled the identifications of putative peptide hormones across different plant species (Yang et al. 2011; Chen et al. 2014; Hou et al. 2014; Wang et al. 2020a; Ali et al. 2024).

Since peptide hormones are believed to be involved in intercellular signaling in plants, a large number of studies have focused on identifying small open reading frames (ORFs) that encode small secreted peptides (SSPs) that are likely to be secreted. In particular, 979 candidate genes were predicated in the genome of *Arabidopsis thaliana*, which encode putative SSPs with lengths between 50 and 150 amino acids (Matsubayashi 2011). Similarly, 1491 putative SSP-encoding genes with 200 or less amino acids were identified in the maize (*Zea mays*) genome (Li et al. 2014b). With genomic sequences for more and more plant species becoming available, research efforts have been made to identify SSPs based on protein properties. A typical SSP precusor protein has a short protein length (often 250 amino acids or less), a N-terminal signal sequence, a conserved peptide-coding motif, and lacking transmembrane regions and endoplasmic reticulum (ER) docking signatures. Furthermore, 4,439 SSP-encoding genes have been found in *Medicago truncatula* (Boschiero et al. 2020), 4,981 in wheat (*Triticum aestivum*) (Tian et al. 2022), and 1,050 in tomato (*Solanaceae lycopersicum*) (Xu et al. 2023b).

When unannotated small ORFs are used in these analyses, much more putative SSPs were predicted in the genomes of different plant species. For example, in *Arabidopsis thaliana*, 33,809 small ORFs encoding putative SSPs were retrieved from its genome through comprehensive annotations and filtrations (Lease and Walker 2006). For poplar (*Populus deltoides*), Yang et al. have identified 12,852 ORFs that encode SSPs ranging from 10 to 200 amino acids (Yang et al. 2011), and a total of 101,048 ORFs likely encoding SSPs were identified in the rice (*Oryza sativa*) genome (Pan et al. 2013). There is a possibility that large numbers of SSPs encoded by non-annotated small ORFs have been ignored in some of the previous studies focusing on annotated proteins. Conversely, results from a recent study in tomato indicated that only a small portion (59) of the 61,306 small ORFs have the characteristics of putative SSPs (Xu et al. 2023b). On the other hand, a large number of the small ORFs predicted might be false positive for SSPs due to lack of strict annotations. Anyway, it is plausible that large numbers of unknown SSPs are to be discovered, and a fraction of them may encode peptide hormones.

To date, more than 30 families of putative peptide hormones have been identified in various plant species, such as CLAVATA3 (CLV3)/EMBRYO SURROUNDING REGION-RELATED peptides (CLEs), C-TERMINALLY ENCODED PEPTIDES (CEPs), PHYTOSULFOKINES (PSKs), EPIDERMAL PATTERNING FACTORS (EPFs), and RAPID ALKALINIZATION FACTORS (RALFs). In addition to typical peptide hormones with an N-terminal secretion peptide signal for the secretory pathway, some putative peptide hormones do not contain an N-terminal signal peptide. These peptides are defined as non-secretory peptides, including SYSTEMINS (SYS), PLANT ELICITOR PEPTIDES (PEPs), ZEA MAYS IMMUNE SIGNALING PEPTIDE 1 (ZIP1), and GRIM REAPER (GRI) PEPTIDE families. Both secreted and non-secretory peptides have been shown to play crucial roles in growth, development and stress response in plants. Detailed information of the identified families of putative peptide hormones and numbers of family members in different plant species are presented in Tables 1 and 2.

**Table 1:** Major SSP families identified in plants

Types	Groups	Families	Descriptions	Bioactive epitopes	Biological functions
Secretory Polypeptide	Pro hydroxylation or glycosylation	CLE	Clavata/Embryo Surrounding Region	13 (CLV3)	Shoot meristem maintenance (CLV3); Lateral root emergence (CLE16/17); Phloem development (CLE33/45).
		IDA/IDL	Inflorescence Deficient in Abscission	14 (IDA)	Floral organ abscission or lateral root emergence (IDA); Pathogen infection or leaf senescence (IDL6).
		CEP	C-terminally Encoded Peptide	15 (AtCEP1)	Primary root growth or lateral root formation (AtCEP1/3); Nitrogen uptake and transport (AtCEP3/5/7).
		PIP/PIPL	PAMP-induced Secreted Peptide	13 (PIP1)	PAMP-induced plant immunity (PIP1); Modulation of salt tolerance (PIP3).
		HYPSYS	Hydroxyproline-rich Systemin	18 (IbHypSys)	Regulation of defense responses (NtHypSys or IbHypSys).
	Tyr sulfation	RGF/GLV/CLEL	Golven/Root Growth Factor/ CLE-Like	13 (GLV6)	Development of root proximal meristem and root gravitropism (RGF1); Pathogen infection reactions(RGF7).
		PSK	Phytosulfokine	5 (AtPSK1)	Cell division, differentiation and expansion (PSK1/5); seed development (GmPSKΓ1).
		PSY	Plant Peptide Containing Sulfated Tyrosine	18 (AtPSY1)	Cell expansion and proliferation, and plant stress responses (PSY1).
		CIF	Casparian Strip Integrity Factor	21 (CIF1)	Formation of Casparian strip (CIF1/2).
	Cys-rich	PDF	Plant Defensin	51 (AtPDF1.2)	Pathogen defense reactions (PDF1.2); Antimicrobial activity (PDF1.1/1.3).
		nsLTP	Non-specific Lipid Transfer Protein	69 (OsLTP2)	Antimicrobial and antifungal activity (TdLTP4/AtLTP4.4); Drought tolerance (TaLTP3).
		NCR	Nodule-specific Cysteine-Rich Peptide	24 (MtNCR247)	Endoreduplication and differentiation (MtNCR247); antimicrobial activity (MtNCR035).
		GASA	Gibberellic Acid Stimulated in Arabidopsis	62 (StSNAKIN)	Fruit maturation and development (FaGAST1); Organ development (FaGAST); Seed germination (GASA4).
		KLP	Knottin-like Peptide	37 (MjAMP1)	Antifungal activity (MjAMP1/2); Insecticidal activity (PA1a/b).
		THL	Thionin-like	43 (AtTHI2.1)	Antifungal and antibacterial Activity (CaThi).
		HLP	Hevein-like Peptide	43 (Hevein)	Antifungal and oomycete pathogens (EAFP1 and EAFP2).
		Cyclotide	Small cyclic peptide	29 (kalata B1)	Anti-HIV activity (kalata B1); Antimicrobial and antifouling activity (Cycloviolacin H4).
		2SA	2 S Albumin	35 (SiAMP2)	Antipathogen responses in (SiAMP2).
		RALF	Rapid Alkalinization Factor	50 (AtRALF23)	Maintenance of pollen tube integrity (AtRALF4/9); Lateral root initiation (AtRALF34); Stress responses (AtRALF22/23).
		HAIRPININS	alfa-HAIRPININ (HAIRPININ)	33 (SmAMP-X)	Broad-spectrum antimicrobial activity (SmAMP-X).
		EPF/EPFL	Epidermal Patterning Factor/EPF-Like	45 (TtEPF9)	Stomatal development (EPF1/2/5/9); Grain size and fertility (OsEPFL5/6).
		PCP	Pollen Coat Protein	59 (PCP-Bγ)	Pollen hydration and germination (PCP-Bγ).
		LURE	LURE-type Cysteine-rich Peptide	62 (TfLURE1)	Pollen tube attraction (AtLURE1 and AtLURE2).
		SCR/SP11	S-locuscysteine-rich Protein/S-locus Protein11	59 (SP11)	Self-incompatibility response and pollen germination (SP11).
		ARACIN	Aracin	40 (AtARACIN1)	Antifungal activities (ARACIN1 and ARACIN2).
		ECL	Egg Cell 1-Like	Uncertain	Double fertilization (EC-1)
		ES	Embryo Sac	61 (ZmES4)	Pollen tube growth and burst (ZmES4).
	Non-Cys, non-PTM	GRP	Glycine-rich protein	Uncertain	Cell lignification (PtGRP1); RNA processing (SaGRP); Components of cell Wall (PvGRP 1.8)
		PNP	Plant Natriuretic Peptide	33 (AtPNP-A)	Plant growth and homeostasis (AtPNP-A). Stomatal movement (irPNP).
		SCOOP	Serine-rich Endogenous Peptide	13 (SCOOP12)	Plant Immunity responses and root growth (SCOOP12).
	Functional Precursor-	CAPE	CAP-derived Peptide	11 (CAPE9)	Salt stress responses, antibacterial and antiherbivory activity (AtCAPE1).
		SUBPEP	Subtilisin-embedded Plant Elicitor Peptide	12 (Gm-SUBPEP)	Defenses against pathogen attack (Gm-SUBPEP).
		INCEPTINS	Inceptins	11 (VuInceptin)	Antiherbivore activity (Inceptins).
Non-Secretory Polypeptide	Immunoreaction	PEP	Plant Elicitor Peptide	23 (AtPEP3)	Modulating innate immunity (AtPEP1); Salt stress responses (AtPEP3); Regenerative responses (REF1).
		ZIP	ZEA MAYS IMMUNE SIGNALING PEPTIDE	17 (ZIP1)	Defenses against pathogenic infaction (ZIP1).
		Sys	Systemin	18 (SlSystemin)	Defenses against herbivorous insects (SlSystemin)
		GRI	GRIM REAPER PEPTIDE	11 (GRI)	Oxidative stress responses (GRI)
	sORF	ENOD40	Early Nodulin 40	13 (MtENOD40-1)	Root nodule initiation and development (ENOD40-1).
		RTFL/DVL	Rotundifolia/Devil	51 (DVL1)	Regulation of cell proliferation and organic development (DVL1 and DVL16).
		PLS	POLARIS	36 (PLS)	Root and leaf vascular development (PLS).
		KOD	KISS OF DEATH	25 (KOD)	Programmed cell death (KOD).
		OSIP	Oxidative Stress-induced Peptide	10 (OSIP108)	Metal stress tolerance (OSIP108).
	microRNA	miPEP	miRNA-Encoded Peptide	33 (miPEP165a)	Root organ growth and development (miPEP165a).

### Classifications of peptide hormones

**Fig. 1:**
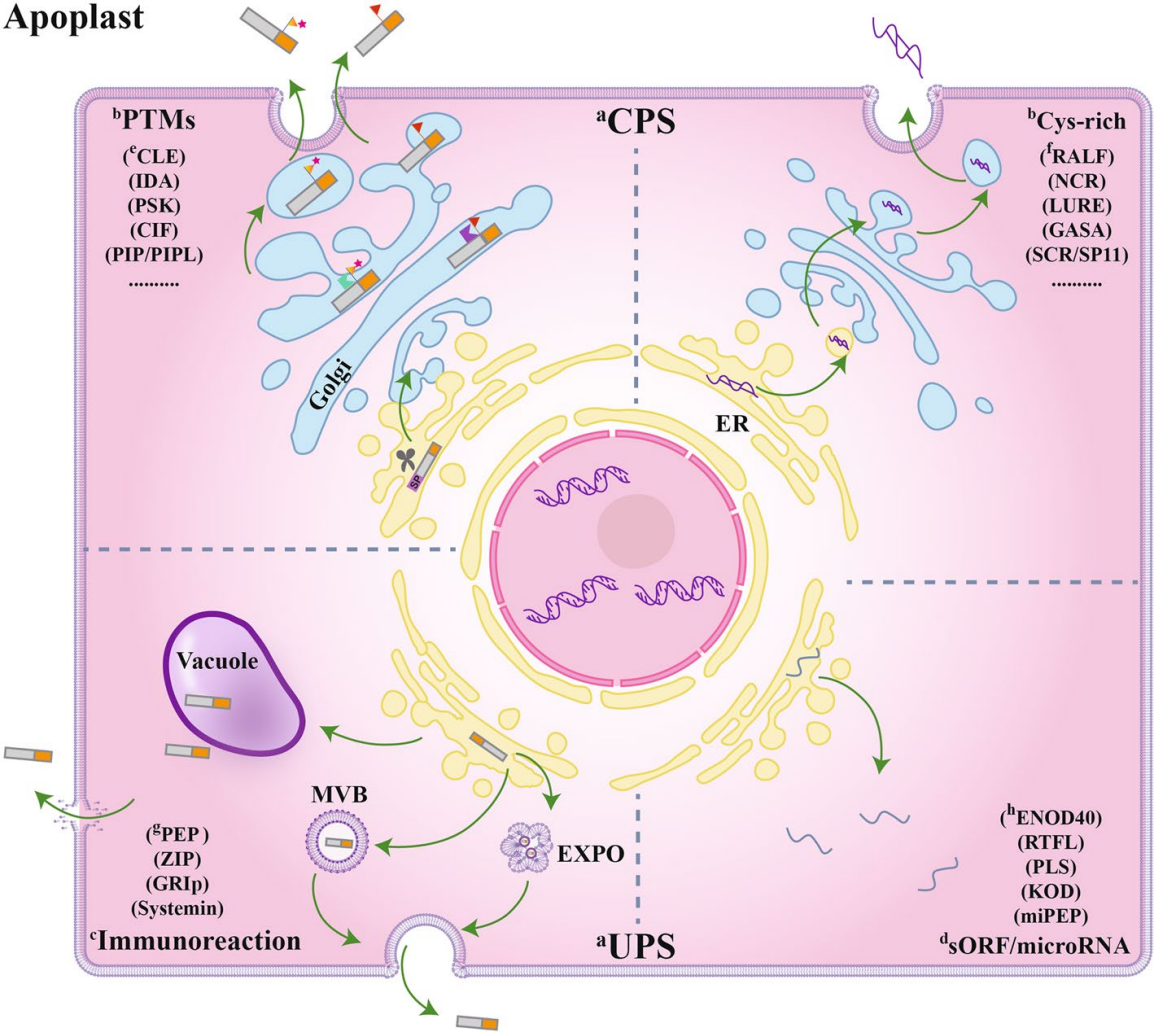
Categorization of peptide hormone families in plants, based on their structures and biosynthetic pathways. Peptide families in plants can be categorized into four distinct groups based on their functions, structures, and biosynthetic pathways. Peptides that undergo post-translational modification (PTM) and exhibit cysteine-richness (Cys-rich) are secreted via the conventional protein secretion (CPS) pathway. In this pathway, preprotein precursors are initially processed in the endoplasmic reticulum (ER), subsequently transported to the Golgi apparatus, and finally secreted into the apoplast through the endomembrane system. In contrast to the CPS pathway, precursors derived from immunoreactive peptides (such as systemin and Pep) and small open reading frames (sORFs) or microRNAs are secreted via the unconventional protein secretion (UPS) pathway, which includes vacuoles, Exocyst-positive organelles (EXPOs), and multivesicular bodies (MVBs). Major references to classification schemes for these polypeptide families are presented, including ^a^CPS and ^a^UPS referenced from Wang X., 2018 (2018); ^b^PTM and ^b^Cys-rich from Matsubayashi 2014 (2014); ^c^Immunoreaction from Del Corpo D., 2024 (2024); ^d^sORF/microRNA from Tavormina P., 2015 (2015); ^e^CLE from Ohyama K., 2009 (2009); ^f^RALF from Murphy E., 2014 (2014); ^g^PEP from Huffaker A., 2006 (2006); ^h^ENOD40 from Campalans A., 2004 (2004)

Peptide hormones in plants can be categorized into different types and families based on criteria including peptide sequences, secretion pathways, functions, origins, biological activities and structural features. In this review, plant peptide hormones are categorized into two types: secretory or non-secretory peptides based on their secretions and localizations in plant cells (Table 1; Fig. 1).

**Table 2:** Number of genes predicted to encode SSPs in different families reported in plants

Peptide families	Numbers of SSP-encoding genes reported	
	Monocotyledonous plants	Dicotyledonous plants
**CLE**	*Oryza sativa*(40)(Gancheva et al. 2021); *Zea mays*(49)(Goad et al. 2017); *Setaria italica*(41)(Ren et al. 2023); *Tricticum aestivum*(104) (Li et al. 2019c).	*Arabidopsis thaliana*(33)(Carbonnel et al. 2023);* Solanum lycopersicum*(52)(Carbonnel et al. 2022); *Medicago truncatula*(52) and *Lotus japonicus*(53)(Hastwell et al. 2017); *Brassica rapa*(29) and *Brassica oleracea*(32) (Xie et al. 2022b); *Glycine max*(84) and *Phaseolus vulgaris*(44)(Hastwell et al. 2015); *Populus trichocarpa*(50)(Han et al. 2016); *Solanum tuberosum*(41)(Gancheva et al. 2021); *Brassica napus*(116)(Han et al. 2020); *Raphanus sativus* (18)(Gancheva et al. 2016); *Vitis vinifera*(9)(Wang et al. 2019); *Cucumis sativus* (26)(Qin et al. 2021); *Thlaspi arvense*(27); *Fagopyrum tataricum*(16) (Hagelthorn and Fletcher 2023); *Gossypium hirsutum*(93)(Wan et al. 2021); *Nicotiana tabacum*(41), *Nicotiana sylvestris*(19) and *Nicotiana tomentosiformis*(24)(Chu et al. 2023).
**CLEL/RGF** **/GLV**	*Zea mays*(14), *Sorghum bicolor*(10), *Setaria italica*(11), *Brachypodium distachyon* (10), *Oryza sativa*(11), *Spirodela polyhiza*(1), *Zostera manna*(1)(Fang et al. 2021); *Tricticum aestivum*(9)(Tian et al. 2022).	***Carica papaya***(5), ***Glycine max***(24), ***Malus domestica***(11), ***Populus trichocarpa***(11) and ***Vitis vinifera***(3)(Fang et al. 2021); ***Solanum lycopersicum*(12)**(Xu et al. 2023b); ***Medicago truncatula*(15)**(De Bang et al. 2017); ***Arabidopsis thaliana*(11)**(Whitford et al. 2012) ; ***Amborella trichopoda*(5)**(Furumizu et al. 2021); ***Aquilegia coerulea*(8)**, ***Ananas comosus*(3)**, ***Brachypodium distachyon*(9) and *Nymphaea colorata*(9)**(Furumizu and Sawa 2021).
**IDA/IDL**	***Zea mays*(3)**(Li et al. 2014b); ***Tricticum aestivum*(18)**(Tian et al. 2022); ***Oil palm*(10)**(Stø et al. 2015).	***Arabidopsis thaliana*(9)**(Vie et al. 2015); ***Citrus clementina*(5)**(Estornell et al. 2015); ***Glycine max*(12) and *Phaseolus vulgaris*(6)**(Tucker and Yang 2012); ***Litchi chinensis*(3)**, ***Lotus japonicus*(1)** and*** Populus trichocarpa*(2)**(Ying et al. 2016); ***Solanum lycopersicum*(8)**(Xu et al. 2023b); ***Fagopyrum tataricum*(3)**(Liu et al. 2021b); ***Medicago truncatula*(42)**(De Bang et al. 2017); ***Lupinus luteus*(1)**(Wilmowicz et al. 2018); ***Nicotiana sylvestris*(5)**, ***Nicotiana tomentosiformis*(5)**, ***Nicotiana tabacum*(10)**, ***Nicotiana benthamiana*(9)**, ***Solanum lycopersicum*(8)**, ***Solanum tuberosum*(7)**, ***Solanum melongena*(6) and* Capsicum annuum*(6)**(Ventimilla et al. 2020).
**CEP**	***Zea mays*(13)**, ***Allium cepa*(1)**, ***Oryza sativa*(9)**, ***Oryza barthii*(1)**, ***Sorghum bicolor*(2)**, ***Triticum Urartu*(2)**, ***Hordeum vulgare*(3)**, ***Saccharum officinarum*(2)**, ***Setaria italica*(1)**, ***Phoenix dactylifera*(5)**, ***Phalaenopsis Aphrodite*(1)**, ***Brachypodium distachyon*(3)**, ***Panicum virgatum*(2)**, ***Aegilops tauschii*(1)**, ***Arachis duranensis*(1) and* Triticum aestivum*(13)**(Delay et al. 2013).	***Arabidopsis thaliana*(15)**, ***Arabidopsis lyrate*(6)**, ***Medicago truncatula*(17)**, ***Gossypium hirsutum*(1)**, ***Lactuca sativa*(4)**, ***Lactuca serriola*(8)**, ***Sesamum indicum*(3)**, ***Arachis hypogaea*(1)**, ***Betula platyphylla*(1)**, ***Siraitia grosvenorii*(1)**, ***Euphorbia esula*(2)**, ***Glycine max*(16)**, ***Lotus japonicus*(7)**, ***Populus trichocarpa*(7)**, ***Vitis vinifera*(8)**, ***Ricinus communis*(8)**, ***Casuarina glauca*(1)**, ***Jatropha curcas*(2)**, ***Theobroma cacao*(4)**, ***Carica papaya*(3)**, ***Fragaria vesca*(3)**, ***Prunus persica*(2)**, ***Manihot esculenta*(2)**, ***Citrus sinensis*(1)**, ***Citrus clementina*(1)**, ***Catharanthus roseus*(1)**, ***Capsella rubella*(2)**, ***Solanum tuberosum*(2)**, ***Brassica napus*(3)**, ***Mimulus guttatus*(3)**, ***Eucalyptus grandis*(2)**, ***Aquilegia coerulea*(1)** and ***Cicer arietinum*(1)**(Delay et al. 2013); ***Malus domestica*(12)**(Yu et al. 2019b); ***Nicotiana tabacum*(21)**(Pan et al. 2024); ***Solanum lycopersicum*(17)**(Liu et al. 2022a); ***Cucumis sativus*(6)**(Liu et al. 2021d); ***Brassica rapa*(27)**(Qiu et al. 2022); ***Lupinus albus*(1)**(Zhou et al. 2019); ***Pisum sativum*(21)**(Lebedeva et al. 2022); ***Fagopyrum tataricum*(5)**(Liu et al. 2021b).
**PIP/PIPL**	***Triticum aestivum*(18)**(Tian et al. 2022); ***Oryza sativa Japonica Group*(1)**, ***Oryza sativa Indica Group*(1)**, ***Sorghum bicolor*(3)**, ***Setaria italica*(1)**, ***Zea mays*(1)**, ***Musa acuminata*(2) and *Brachypodium distachyon*(1)**(Yu et al. 2023b).	***Arabidopsis thaliana*(11)**(Yu et al. 2023b); ***Medicago truncatula*(13)**(De Bang et al. 2017); ***Fagopyrum tataricum*(1)**(Liu et al. 2021b); ***Solanum lycopersicum*(4)**(Xu et al. 2023b); ***Solanum tuberosum*(4)**(Combest et al. 2021); ***Brassica oleracea*(13)**, ***Brassica rapa*(13)**, ***Brassica napus*(27)**, ***Nicotiana attenuate*(8)**, ***Phaseolus vulgaris*(8)**, ***Vigna radiata*(3)**, ***Glycine max*(14)**, ***Cucumis sativus*(4) and *Cucumis melo*(6)**(Yu et al. 2023b).
**PSK**	***Oryza sativa*(7)**(Yang et al. 1999); ***Zea mays*(7)**(Li et al. 2014b); ***Setaria italica*(1)**(Wu et al. 2019); ***Asparagus officinalis*(2)**(Yang et al. 2001); ***Triticum aestivum*(15)**(Tian et al. 2022); ***Sorghum bicolor*(1) and *Sorghum propinquum*(1)**(Di et al. 2022).	***Arabidopsis thaliana*(9)**(Stührwohldt et al. 2021); ***Pyrus bretschneideri*(10)**, ***Malus × domestica*(11)**, ***Prunus persica*(4)**, ***Fragaria vesca*(6) and *andPrunus mume*(5)**(Kou et al. 2020); ***Populus trichocarpa*(2)**(Wu et al. 2019); ***Solanum lycopersicum*(8)**(Xu et al. 2023b); ***Fagopyrum tataricum*(5)**(Liu et al. 2021b); ***Hevea brasiliensis*(6)**(Gao et al. 2023); ***Lotus japonicas*(5)**(Wang et al. 2015a); ***Zinnia elegans*(1)**(Motose et al. 2009); ***Daucus carota*(1)**(Hanai et al. 2000); ***Gossypium hirsutum*(2)**(Han et al. 2014); ***Brassica napus*(1)**, ***Gossypium arboretum*(1)**, ***Lycopersicon esculentum*(4)**, ***Mesembryanthemum cristallinum*(1)** and ***Solanum tuberosum*(3)**(Lorbiecke and Sauter 2002); ***Lotus japonicus*(7)**, ***Glycine max*(19) and *Medicago truncatula*(7)**(Di et al. 2022).
**PSY**	***Zea mays*(8)**(Tost et al. 2021); ***Triticum aestivum*(29)**(Tian et al. 2022); ***Oryza sativa*(7)**(Kesawat et al. 2024).	***Arabidopsis thaliana*(8)**, ***Amborella trichopoda*(1)**, ***Glycine max*(3)**, ***Cicer arietinum*(1)**, ***Lotus japonicus*(1)**, ***Populus trichocarpa*(1)** and ***Medicago truncatula*(1)**(Tost et al. 2021); ***Solanum lycopersicum*(11)**(Xu et al. 2023b).
**CIF**	***Triticum aestivum*(8)**(Tian et al. 2022).	***Arabidopsis thaliana*(5)**(Fujita 2021); ***Fagopyrum tataricum*(2)**(Liu et al. 2021b).
**HYPSYS**	***Ipomoea batatas*(1)**(Chen et al. 2008).	***Nicotiana tabacum*(1)**(Pearce et al. 2001a); ***Solanum lycopersicum*(1)**(Pearce and Ryan 2003); ***Petunia hybrida*(2)**(Pearce et al. 2007); ***Solanum tuberosum*(1)**(Bhattacharya et al. 2013); ***Solanum nigrum*(1)**(Pearce et al. 2009).
**RALF**	***Oryza sativa*(43) and *Zea mays*(34)**(Sharma et al. 2016);***Ananas comosus*(14)**, ***Brachypodium distachyon*(10)**, ***Brachypodium stacei*(11)**, ***Musa acuminata*(13)**, ***Panicum hallii*(13)**, ***Panicum virgatum*(31)**, ***Setaria italica*(15)**, ***Setaria viridis*(1)**, ***Sorghum bicolor*(16) and *Spirodela polyrhiza*(1)**(Campbell and Turner 2017); ***Saccharum spp.*(4)**(Mingossi et al. 2010); ***Triticum aestivum*(38)**(Tian et al. 2022).	***Arabidopsis thaliana*(39) and *Glycine max*(18)**(Sharma et al. 2016); ***Amaranthus hypochondriacus*(12)**, ***Amborella trichopoda*(9)**, ***Aquilegia coerulea*(12)**, ***Arabidopsis helleri*(25)**, ***Arabidopsis lyrate*(33)**, ***Brassica rapa*(32)**, ***Capsella grandiflora*(24)**, ***Capsella rubella*(33)**, ***Carica papaya*(17)**,***Citrus sinensis*(14)**, ***Citrus clementina*(13)**, ***Eucalyptus grandis*(16)**, ***Eutrema salsugineum*(35)**, ***Gossypium raimondi*(33)**, ***Linum usitatissimum*(20)**, ***Medicago truncatula*(13)**, ***Mimulus guttatus*(17)**, ***Phaseolus vulgaris*(9)**, ***Prunus persica*(14)**, ***Ricinus communis*(18)**, ***Solanum tuberosum*(16)**, ***Theobroma cacao*(13) and *Vitis vinifera*(4)**(Campbell and Turner 2017); ***Solanum chacoense*(5)**(Germain et al. 2005); ***Lotus japonicus*(17) and *Medicago truncatula*(12)**(Jia and Li 2023); ***Populus trichocarpa*(23)**(Cao and Shi 2012); ***Prunus mume*(9)**, ***Prunus avium*(17)**, ***Pyrus communis*(10)**, ***Fragaria vesca*(13)**(Zhang et al. 2020); ***Lycopersicon esculentum*(1)**(Scheer et al. 2005); ***Broccoli flowers*(1)**(Zhang et al. 2010); ***Gossypium arboretum*(42)**, ***Gossypium raimondi*(38)**, ***Gossypium hirsutum*(104) and *Gossypium barbadense*(120)**(Lin et al. 2022); ***Cucumis sativus*(17)**(Kiryushkin et al. 2023); ***Fagopyrum tataricum*(10)**(Liu et al. 2021b); ***Nicotiana tabacum*(1)**(Pearce et al. 2001b); ***Malus domestica*(33)**(Campbell and Turner 2017).
**SNAKIN** **/GASA**	***Triticum aestivum*(37)**(Tian et al. 2022); ***Zea mays*(12)**(Li et al. 2014b); ***Phyllostachys edulis*(8)**(Hou et al. 2018); ***Sorghum bicolor*(12)**(Filiz and Kurt 2020); ***Sorghum bicolor*(12) and* Brachypodium distachyon*(11)**(Panji et al. 2023); ***Oryza sativa*(13) and *Setaria itali*c (7)**(Muhammad et al. 2019); ***Ananas comosus*(15); *Hordeum vulgare*(11); *Triticum turgidum*(19)**(Bouteraa et al. 2023).	***Citrus clementina*(18)**(Wu et al. 2021); ***Arabidopsis thaliana*(15)**(Zhang and Wang 2008); ***Hevea brasiliensis*(16)**(An et al. 2018); ***Prunus mume*(16)**(Zhang et al. 2022c); ***Solanum lycopersicum*(20)**(Xu et al. 2023b); ***Fagopyrum tataricum*(20)**(Liu et al. 2021b); ***Glycine max*(37)**(Zulfiqar et al. 2019); ***Populus trichocarpa*(21)**(Wu et al. 2022); ***Populus euphratica*(19)**(Han et al. 2021); ***Solanum tuberosum*(16)**(Nahirñak et al. 2016); ***Theobroma cacao*(17)**(Abdullah et al. 2021); ***Nicotiana tabacum*(18)**(Li et al. 2022d); ***Cucumis sativus*(9)**, ***Citrullus lanatus*(9)**, ***Cucumis melo*(10)**, ***Cucurbita moschata*(11)**, ***Benincasa hispida*(9)**, ***Luffa cylindrica*(9)**, ***Lagenaria siceraria*(8)**, ***Momordica charantia*(15)**, ***Sechium edule*(16) and* Trichosanthes anguina*(18)**(Zhang et al. 2023); ***Brassica rapa pekinensis*(15)**(Sun et al. 2023); ***Solanum lycopersicon*(19) and* Capsicum annuum*(10)**(Muhammad et al. 2019); ***Vitis vinifera*(14)**(Ahmad et al. 2020); ***Arachis hypogaea*(9)**(Syed Nabi et al. 2023); ***Lactuca sativa*(20)**(Biology et al. 2023);***Malus domestica*(26)**(Fan et al. 2017); ***Gossypium herbaceum*(19)**, ***Gossypium arboretum*(17)**, ***Gossypium raimondii*(25)**, ***Gossypium barbadense*(33) and* Gossypium hirsutum*(38)**(Qiao et al. 2021); ***Canavalia rosea*(23)**(Zhang et al. 2022d); ***Phaseolus vulgaris*(23)**(Bouteraa et al. 2023).
**PCP**	***Oryza sativa*(2)**, ***Hordeum vulgare*(26)**, ***Triticum urartu*(4)**, ***Aegilops tauschii*(4)**, ***Brachypodium distachyon*(2)**, ***Zea mays*(12)**, ***Sorghum bicolor*(14)**, ***Panicum virgatum*(3)**, ***Panicum hallii*(3)**, ***Setaria italica*(8)**, ***Oropetium thomaeum*(2) and* Eragrostis tef*(11)**(Wang et al. 2017a).	***Arabidopsis thaliana*(11)**, ***Brassicarapa*(20)**, ***Brassica juncea*(18) and *Brassica oleracea*(16)**(Liu 2024); ***Capsella rubella*(10)**, ***Capsella grandiflora*(12)**, ***Capsella orientalis*(13)**, ***Neslia paniculata*(3)**, ***Camelina sativa*(4)**, ***Leavenworthia alabamica*(4)**, ***Arabis alpina*(3)**, ***Brassica napus*(16)**, ***Raphanus raphanistrum*(6)**, ***Raphanus sativus*(9)**, ***Sisymbrium irio*(3)**, ***Eutrema salsugineum*(7)**, ***Tarenaya hassleriana*(8)**, ***Gossypium arboreum*(1)**, ***Nelumbo nucifera*(14)**, ***Sesamum indicum***,*** Nicotiana benthamiana *and *Mimulus guttatus*(2)**(Wang et al. 2017a).
**EPF/EPFL**	***Oryza sativa*(11)**(Xiong et al. 2022); ***Sorghum bicolor*(12)**(Jiao et al. 2023); ***Triticum aestivum*(29)**(Tian et al. 2022); ***Zea mays*(8)**(Li et al. 2014b); ***Secale cereale*(12)**(Zhiling et al. 2024).	***Arabidopsis thaliana*(11)**, ***Carica papaya*(10)** and ***Medicago truncatula*(12)**(Takata et al. 2013); ***Brassica napus*(2)**(Huang et al. 2014); ***Fagopyrum tataricum*(11)**(Liu et al. 2021b); ***Solanum lycopersicum*(12)**(De Bang et al. 2017); ***Populus trichocarpa*(15)**(Liu et al. 2016).
**nsLTP**	***Oryza sativa*(82)**, ***Sorghum bicolor (*63) and** ***Zea mays*(63)**(Fonseca-García et al. 2021); ***Sorghum spontaneum*(7)**(de Oliveira Silva et al. 2022); ***Triticum aestivum*(463)**(Tian et al. 2022); ***Setaria italica*(45)**, ***Brachypodium distachyon*(30) and* Setaria viridis*(45)**(Li et al. 2022a); ***Hordeum vulgare*(70)**(Zhang et al. 2019b); ***Saccharum spontaneum*(21)**(de Oliveira Silva et al. 2022).	***Arabidopsis thaliana*(79)**(Fleury et al. 2019); ***Lotus japonicus*(72)**, ***Phaseolus vulgaris*(77)**, ***Glycine max*(120)**, ***Medicago truncatula*(95)**, ***Trifolium pratense*(85)**, ***Lupinus albus*(87) and *Pisum sativum*(73)**(Fonseca-García et al. 2021); ***Brassica napus*(246)**(Liang et al. 2022); ***Helianthus annuus*(101)**(Vangelisti et al. 2022); ***Sesamum indicum*(52)**(Song et al. 2021a); ***Arachis duranensis*(64)**(Song et al. 2020); ***Solanum tuberosum*(83)**(Li et al. 2019a); ***Solanum lycopersicum*(122)**(Xu et al. 2023b); ***Gossypium arboretum*(51)**, ***Gossypium raimondii*(47) and *Gossypium hirsutum*(91)**(Li et al. 2016); ***Brassica rapa*(63)**(Li et al. 2014a); ***Lotus japonicus*(24)**(Tapia et al. 2013); ***Capsicum annuum*(19)**, ***Nicotiana benthamiana*(17)and *Petunia hybrida*(10)**(Liu et al. 2010); ***Nicotiana tabacum*(100)**, ***Nicotiana sylvestris*(50) and *Nicotiana tomentosiformis*(51)**(Yang et al. 2022); ***Lactuca sativa*(105)**, ***Manihot esculenta*(98)**, ***Mimulus guttatus*(114)**, ***Sinapis alba*(189) and *Spinacea oleracea*(43)**(Santos-Silva et al. 2023); ***Fagopyrum tataricum*(36)**(Liu et al. 2021b); ***Aquilegia coerulea*(10)**(Wang et al. 2012);***Coffea arabica*(4) and *Coffea canephora*(3)**(Cotta et al. 2014); ***Theobroma cacao*(46)**(Fleury et al. 2019); ***Populus trichocarpa*(93)**(Wei et al. 2022).
**PDF**	***Avena sativa*(1)**(Emamifar et al. 2021); ***Hordeum vulgare*(3)**(Thomma et al. 2002); ***Triticum aestivum*(80)**(Tian et al. 2022); ***Zea mays*(4)**(Cordts et al. 2001); ***Sorghum bicolor*(6)**(Thomma et al. 2002); ***Oryza sativa*(7)**(Tantong et al. 2016); ***Elaeis guineensis*(1) and *Triticum kiharae*(7)**(van der Weerden and Anderson 2013).	***Arabidopsis thaliana*(15)**(Thomma et al. 2002); ***Arachis hypogaea*(12)**(Zhao et al. 2022); ***Brassica napus*(37)**(Liu et al. 2021c); ***Arabidopsis helleri*(4) and *Raphanus sativus*(4)**(Mirouze et al. 2006); ***Petunia hybrida*(2)**(Lay et al. 2003); ***Dahlia merkii*(1)**, ***Aesculus hippocastanum*(1) and *Clitoria ternatea*(1)**(Thevissen et al. 2000); ***Cicer arietinum*(16)**(Nitnavare et al. 2023); ***Gerbera hybrida*(9)**(Cheng et al. 2024); ***Medicago truncatula*(63)**(Velivelli et al. 2018); ***Carthamus tinctorius*(1)**, ***Brassica juncea*(1) and *Nicotiana alata*(2)**(Cheng et al. 2024); ***Solanum lycopersicum*(51)**(Xu et al. 2023b); ***Fagopyrum tataricum*(16)**(Liu et al. 2021b); ***Aesculus Beta vulgaris*(2)**, ***Brassica rapa*(1)**, ***Capsicum annuum*(3)**, ***Capsicum chinense*(1)**, ***Cassia fistula*(2)**, ***Citrus paradisi*(1)**, ***Glycine max*(3)**, ***Helianthus annuus*(4)**, ***Heuchera sanguinea*(1)**, ***Lycopersicon esculentum*(2)**, ***Nicotiana excelsior*(2)**, ***Nicotiana tabacum*(3)**, ***Petunia integrifolia*(1)**, ***Phaseolus coccineus*(1)**, ***Pisum sativum*(4)**, ***Pyrus pyrifolia*(2)**, ***Raphanus sativus*(4)**, ***Solanum tuberosum*(1)**, ***Spinacia oleracea*(1)**, ***Vicia faba*(2) and *Wasabia japonica*(1)**(Thomma et al. 2002); ***Arachis diogoi*(2)**, ***Pentadiplandra brazzeana*(1)**, ***Brassica campestris*(1)**, ***Cajanus cajan*(1)**, ***Vigna unguiculata*(3)**, ***Ginkgo biloba*(1)**, ***Heliophila coronopifolia*(2)**, ***Hardenbergia violacea*(1)**, ***Lepidium meyenii*(1)**, ***Medicago sativa*(4)**, ***Nicotiana attenuate*(1)**, ***Nicotiana paniculata*(2)**, ***Brassica oleracea*(3)**, ***Pachyrhizus erosus*(1)**, ***Plantago major*(1)**, ***Petunia inflat*(1)**, ***Sinapis alba*(3)**, ***Trigonella foenum-graecum*(1)**, ***Tephrosia platycarpa*(1)**, ***Tephrosia villosa*(1)**, ***Vigna angularis*(1)**, ***Vigna radiata*(2)and *Wasabi japonica*(1)**(van der Weerden and Anderson 2013).
**HLPs**	***Oryza sativa*(1)**(Porto et al. 2012); ***Triticum aestivum*(47)**(Tian et al. 2022); ***Triticum kiharae*(3) and *Triticum timopheevii*(2)** (Andreev et al. 2012);***Aegilops speltoides*(1)**, ***Aegilops Searsii*(1)**, ***Aegilops Mutica*(4)**, ***Aegilops Caudata*(5)**, ***Aegilops Cylindrica*(1)**, ***Aegilops ventricosa*(1)**, ***Aegilops Columnaris*(1)**, ***Aegilops Juvenalis*(1)**, ***Aegilops Recta (*1) and *Aegilops tauschii*(1)**(Istomina et al. 2017); ***Leymus arenarius*(1)**(Utkina et al. 2010); ***Elytrigia repens*(3)**(Slezina et al. 2018).	***Arabidopsis thaliana*(10) and *Medicago truncatula*(15)**(Zhou et al. 2013); ***Solanum lycopersicum*(9)**(Xu et al. 2023b); ***Hevea brasiliensis*(1)**(ARCHER 1960); ***Pharbitis nil*(2) and *Fagopyrum esculentum*(3)**(Fujimura et al. 2003); ***Vitis vinifera*(1)**(Porto et al. 2012); ***Ginkgo biloba*(11)**(Wong et al. 2016); ***Amaranthus caudatus*(2)**(Broekaert et al. 1992); ***Eucommia ulmoides*(2)**(Xiang et al. 2002);***Alternanthera sessilis*(6)**(Kini et al. 2015); ***Chenopodium quinoa*(3)**(Loo et al. 2021); ***Amaranthus retroflexus*(1)**(Lipkin et al. 2005); ***Beta vulgaris*(1)**(Nielsen et al. 1997); ***Stellaria media*(14)**, ***Dianthus caryophyllus*(3) and* Silena latifolia*(6)**(Slavokhotova et al. 2017); ***Amaranthus hypochondriacus*(1)**(Rivillas-Acevedo and Soriano-García 2007); ***Eucommia ulmoides*(2)**(Huang et al. 2002); ***Euonymus europaeus*(1)**(Van den Bergh et al. 2002); ***Vaccaria hispanica*(2)**(Wong et al. 2017); ***Moringa oleifera*(4)**(Márjory et al. 2020); ***Capsicum annuum*(1)**(Games et al. 2016); ***Wasabia japonica*(1)**(Kiba et al. 2003); ***Broussonetia Papyrifera***,*** syn. Morus papyrifera*(2)**(Zhao et al. 2011).
**2 S Albumins**	***Triticum aestivum*(2)**(Tian et al. 2022); ***Passiflora alata*(1)**(Ribeiro et al. 2011).	***Solanum lycopersicum*(2)**(Xu et al. 2023b); ***Brassica napus*(2)**, ***helianthus annuus*(1)**, ***leonurus japonicus*(1)**, ***Sesamum indicum*(1)**, ***Momordica charantia*(1)**, ***Mirabilis jalapa*(1) and *Passiflora edulis*(1)**(Maria-Neto et al. 2011); ***Ricinus communis*(7)**(Do Nascimento et al. 2011); ***Arachis hypogaea*(1)**(Duan et al. 2013); ***Juglans regia*(1)**(Sordet et al. 2009); ***Raphanus sativus*(2)**(Terras et al. 1992); ***Passiflora edulis f. flavicarp*(2)**(Agizzio et al. 2003); ***Cicer arietinum*(1)**(Vioque et al. 1999); ***Helianthus annuus*(1)**(Regente and De la Canal 2001); ***Brassica juncea*(1)**(Mandal et al. 2002); ***Cucurbita moschata*(1)**(Souza 2020); ***Capsicum annuum*(1)**(Ribeiro et al. 2012); ***Jatropha curcas*(1)**(Turner 2014); ***Pistacia vera*(1)**(Taghizadeh et al. 2020).
**α-Hairpinin**	***Triticum kiharae*(2)**(Utkina et al. 2013); ***Triticum aestivum*(4)**(Tian et al. 2022); ***Zea mays*(1)**(Duvick et al. 1992); ***Echinochloa cruss-galli*(2)**(Souza et al. 2014).	***Fagopyrum esculentum*(3)**(Park et al. 1997); ***Veronica hederifolia*(1)**(Conners et al. 2007); ***Cucurbita maxima*(1)**(Yamada et al. 1999); ***Macadamia integrifolia*(1)**, ***Luffa aegyptiaca*(1) and *Stellaria media*(1)**(Souza et al. 2014).
**KTPs**		***Mirabilis jalapa*(2)**(Cammue et al. 1992); ***Phytolacca americana*(1)**(Gao et al. 2001); ***Pisum sativum*(1)**(Chouabe et al. 2011); ***Hibiscus sabdariffa*(8)**(Loo et al. 2016).
**THL**	***Oryza sativa*(44)**(Silverstein et al. 2007); ***Triticum aestivum*(32)**(Tian et al. 2022); ***Arena sativa*(2*) *and *Secale cereale*(1)**(Florack and Stiekema 1994); ***Hordeum vulgare*(5)**, ***Hordeum murinum*(6)**, ***Hordeum marinum*(1) and *Hordeum jubatum*(1)**(Bunge et al. 1992); ***Hordeum jubatum*(1)**(Schrader and Apel 1991); ***Hordeum wulgare*(1)**, ***Tulipa gesneriana*(1)**, ***Panicum miliaceum*(1)**, ***Eleusine coracana*(1)**, ***Neurachne alopecuroidea*(4)**,***Neurachne munroi*(1)**, ***Thyridolepis multiculmis*(2)**, ***Thyridolepis mitchelliana*(3) and *Neurachne lanigera*(1)**(Höng et al. 2021).	***Solanum lycopersicum*(18)**(Xu et al. 2023b); ***Arabidopsis thaliana*(71)**(Almaghrabi et al. 2019); ***Capsicum annuum*(5)**(Taveira et al. 2014); ***Nigella sativa*(9)**(Barashkova et al. 2021); ***Phoradendron tomentosum*(1) and* Crambe abyssinica*(2)**(Florack and Stiekema 1994); ***Viscum album*(2)**(Schrader and Apel 1991); ***Crambe abyssinica*(1)**, ***Oresitrophe rupifraga*(3)**, ***Viola tricolor*(1)**, ***Papaver rhoeas*(14)**, ***Papaver somniferum*(9)**, ***Papaver setigerum*(6)**, ***Papaver bracteatum*(9)**, ***Sassafras albidum*(1)**, ***Lindera benzoin*(1)**, ***Chrysobalanus icaco*(1)**,***Thalictrum thalictroides*(3)**, ***Draba sachalinensis*(2)**, ***Myristica fragrans*(2)**, ***Mirabilis jalapa*(1)**, ***Hydrocotyle umbellate*(1)**, ***Prunella vulgaris*(1)**, ***Trianthema portulacastrum*(5)**, ***Urtica dioica*(2)**, ***Brassica nigra*(3)**, ***Arabis alpina*(6)**, ***Sinapis alba*(3)**, ***Draba hispida*(1)**, ***Draba oligosperma*(1)**, ***Draba aizoides*(1)**, ***Draba magellanica*(1)**, ***Aerva lanata*(1) and* Portulaca mauii*(1)**(Höng et al. 2021).
**LURE**		***Arabidopsis thaliana*(8) and* Arabidopsis lyrata*(10)**(Takeuchi 2021); ***Torenia fournieri*(2) **(Okuda et al. 2009);***Torenia concolor*(1)**(Kanaoka et al. 2011).
**NCR**		***Medicago truncatula*(639)**, ***Medicago sativa*(469)**, ***Galega orientalis*(313)**, ***Ononis spinosa*(234)**, ***Onobrychis viciifolia*(171)**, ***Astragalus canadensis*(108)**, ***Cicer arietinum*(63)**, ***Oxytropis lamberti*(36) and *Glycyrrhiza uralensis*(7)**(Montiel et al. 2017); ***Astragalus sinicus*(7)**(Chou et al. 2006); ***Vicia faba*(5)**(Frühling et al. 2000); ***Pisum sativum*(360)**(Zorin et al. 2022); ***Aeschynomene afraspera*(38)**, ***Aeschynomene indica*(44) and* Aeschynomene evenia*(82)**(Czernic et al. 2015); ***Trifolium repens*(1)**(Crockard et al. 2002); ***Arabidopsis thaliana*(3)**(Zhou et al. 2013).
**CYCLOTIDE**		***Viola abyssinica*(6)**(Yeshak et al. 2011); ***Viola arvensis*(9)**, ***Viola decumbens*(1)**,***Viola hederacea*(7) and *Viola nivalis*(1)**(Gerlach and Mondal 2012); ***Viola odorata*(30)**(Ireland et al. 2006); ***Viola biflora*(11)**(Herrmann et al. 2008); ***Viola baoshanensis*(23)**(Zhang et al. 2009); ***Viola cotyledon*(2)**(Göransson et al. 2003); ***Viola labridorica*(8)**(He et al. 2011a); ***Viola ignobilis*(6)**(Farhadpour et al. 2016); ***Viola philippica*(16)**(He et al. 2011b); ***Viola tricolor*(14)**(Tang et al. 2010); ***Viola yedoensis*(8)**(Wang et al. 2008);***Gloeospermum blakeanum*(7) and *Gloeospermum pauciflorum*(7)**(Burman et al. 2010); ***Hybanthus parviflorus*(1)**(Broussalis et al. 2001); ***Noisettia orchidiflora*(1)**(De Veer et al. 2019); ***Melicytus chathamicus*(7) and *Melicytus latifolius*(7)**(Ravipati et al. 2015); ***Hybanthus floribundus*(11)**, ***Hymanthera obovate*(1)**, ***Melicytus ramiflorus*(3)**, ***Melicytus macrophyllus*(2)**, ***Rinorea gracilipes*(1)*Rinorea lindeniana*(2)**, ***Chassalia discolor*(1)**, ***Chassalia parvifolia*(6)**, ***Oldenlandia affinis*(18)**, ***Psychotria leptothyrsa*(6)**, ***Psychotria suterella*(1)**, ***Psychotria poeppigiana*(1)**, ***Clitoria ternatea*(24)**,(Gerlach et al. 2013); ***Pombalia calceolaria*(2)**(Pinto et al. 2018); ***Rinorea bengalensis*(1) and *Rinorea Virgate*(7)**(Niyomploy et al. 2018); ***Rinorea dentate*(1)**(Attah et al. 2016); ***Rinorea sumatrana*(4)**(Niyomploy et al. 2016); ***Leonia cymose*(4)**(Hallock et al. 2000); ***Carapichea ipecacuanha*(14)**(Fahradpour et al. 2017); ***Chassalia chartacea*(18)**(Nguyen et al. 2012); ***Palicourea condensate*(1)**(Bokesch et al. 2001); ***Palicourea rigida*(3)**(Pinto et al. 2016); ***Psychotria leiocarpa*(5) and *Psychotria brachyceras*(7)**(Matsuura et al. 2016); ***Hedyotis biflora*(2)**(Nguyen et al. 2011); ***Psychotria longipes*(1)**(Witherup et al. 1994); ***Psychotria solitudinum*(1)**(Hellinger et al. 2015); ***Momordica cochinchinensis*(3)**(Hernandez et al. 2000); ***Momordica diocia*(6)**(Du et al. 2019); ***Momordica macrophylla*(2)**, ***Momordica anigosantha*(1)**, ***Momordica subangulata*(1)**, ***Momordica sphaeroidea*(1)**, ***Momordica clarkeana*(1)**, ***Momordica denticulate*(1) and *Momordica gilgiana*(1)**(Mahatmanto et al. 2015).
**SYS**		***Solanum lycopersicum*(1)**(Pearce et al. 1991); ***Solanum tuberosum*(2)**, ***Solanum nigrum*(1) and *Capsicum annuum*(1)**(Constabel et al. 1998).
**SCOOP**		***Arabidopsis thaliana*(50)**(Yang et al. 2023).
**PEP**	***Brachypodium distachyon*(1)**, ***Zea mays*(7)**, ***Oryza brachyantha*(1)**, ***Oryza sativa*(3)**, ***Sorghum bicolor*(3) and *Setaria italica*(2)**(Lori et al. 2015).	***Arabidopsis thaliana*(8)**(Bartels et al. 2013); ***Glycine max*(6)**(Lee et al. 2018); ***Brassica oleracea*(9)**(Wang et al. 2022a); ***Solanum lycopersicum*(1)**(Yang et al. 2024); ***Arabidopsis arenosa*(4)**, ***Aquilegia coerulea*(1)**, ***Arabidopsis lyrate*(6)**, ***Brassica napus*(1)**, ***Brassica rapa*(5)**, ***Cicer arietinum*(1)**, ***Capsella rubella*(5)**, ***Citrus sinensis*(1)**,***Eutrema salsugineum*(4)**, ***Gossypium arboretum*(1)**, ***Morus notabilis*(1)**, ***Medicago truncatula*(2)**, ***Nicotiana benthamiana*(1)**, ***Nicotiana sylvestris*(1)**, ***Nicotiana tomentosiformis*(1)**, ***Prunus mume*(1)**, ***Populus trichocarpa*(1)**, ***Phaseolus vulgaris*(1)**, ***Ricinus communis*(2)**, ***Solanum melongena*(1)**, ***Solanum tuberosum*(1)**, ***Theobroma cacao*(1)**, ***Vitis vinifera*(1)**, ***Brachypodium distachyon*(1)**, ***Oryza brachyantha*(1)**, ***Oryza sativa*(3)**, ***Sorghum bicolor*(3) and *Setaria italica*(2)**(Lori et al. 2015); ***Malus domestica*(2)**, ***Fragaria ananassa*(2)**, ***Fragaria vesca*(1)**, ***Prunus avium*(2)**, ***Prunus dulcis*(2)**, ***Prunus domestica*(2)**, ***Prunus mume*(2)**, ***Prunus persica*(2)**, ***Prunus nucipersica*(2)**,** and *Pyrus bretschneideri*(1)**(Ruiz et al. 2018).
**CAPE**	***Triticum aestivum*(79)**(Tian et al. 2022); ***Musa acuminata*(13) and *Musa balbisiana*(10); *Triticum durum*(9); *Hordeum vulgare*(11)**(Yin et al. 2023); ***Zea mays*(1) and *Oryza Sativa*(1)**(Chen et al. 2014);	***Arabidopsis thaliana*(9)**(Chien et al. 2015); ***Fagopyrum tataricum*(28)**(Liu et al. 2021b); ***Solanum lycopersicum*(13)**(Akbudak et al. 2020); ***Camellia sinensis*(16)**(Zhang et al. 2022e); ***Glycine max*(21)**(Almeida-Silva and Venancio 2022); ***Piper nigrum*(8)**(Kattupalli et al. 2021); ***Populus trichocarpa*(12)**(Wang et al. 2023b); ***Capsicum annuum*(1)**, ***Solanum tuberosum*(1)**, ***Capsicum frutescens*(2)**, ***Solanum phureja*(1)**, ***Nicotiana glutinosa*(1)**, ***Nicotiana tabacum*(1)**, ***Vitis vinifera*(1)**, ***Vitis hybrid cultivar*(1)**, ***Vitis shuttleworthii*(1)**, ***Brassica napus*(1)**, ***Brassica rapa*(1) and *Medicago truncatula*(1)**(Chen et al. 2014).
**INCEPTINS**	***Zea mays*(1)**, ***Tricticum aestivum*(1)**, ***Sorghum bicolor*(1) and *Oryza sativa*(1)**(Schmelz et al. 2006).	***Vigna unguiculata*(1)**, ***Glycine max*(1)**, ***Phaseolus vulgaris*(1)**, ***Arachis hypogaea*(1)**, ***Phaseolus lunatus*(1)**, ***Brassica oleracea*(1)**, ***Arabidopsis thaliana*(1)**, ***Solanum tuberosum*(1)**, ***Cicer arietinum*(1)**, ***Lens culinaris*(1)**, ***Pisum sativum*(1)**, ***Medicago truncatula*(1)**, ***Lycopersicon esculentum*(1) and *Nicotiana tabacum*(1)**(Schmelz et al. 2006).
**SUBPEP**	***Triticum aestivum*(12)**(Tian et al. 2022).	***Medicago truncatula*(1)**(De Bang et al. 2017); ***Glycine max*(1)**(Pearce et al. 2010); ***Solanum lycopersicum*(3)**(Xu et al. 2023b).
**ENOD40**	***Oryza sativa*(2)**, ***Oryza brachyantha*(2)**, ***Oryza meyeriana*(2)**, ***Leersia perrieri*(2)**, ***Zizania latifolia*(2)**, ***Triticum aestivum*(4)**, ***Aegilops tauschii*(1)**, ***Thinopyrum elongatum*(1)**, ***Secale cereale*(1)**, ***Hordeum vulgare*(1)**, ***Lolium perenne*(1)**, ***Dactylis glomerata*(1)**, ***Puccinellia tenuiflora*(1)**, ***Brachypodium distachyon*(1)**, ***Phyllostachys edulis*(4)**, ***Raddia distichophylla*(2)**, ***Dendrocalamus latiflorus*(2)**, ***Zea mays*(2)**, ***Sorghum bicolor*(2)**, ***Saccharum officinarum*(2)**, ***Panicum virgatum*(4)**, ***Panicum miliaceum*(5)**, ***Setaria italica*(2)**, ***Eragrostis curvula*(2)**, ***Zoysia japonica*(2)**, ***Eleusine indica*(2) and *Oropetium thomaeum*(2)**(Gultyaev et al. 2023); ***Festuca arundinacea*(1)**, ***Leymus chinensis*(1) and *Avena sativa*(1)**(Gultyaev and Roussis 2007).	***Medicago truncatula*(2)**(De Bang et al. 2017); ***Lotus japonicus*(2*)***(Kumagai et al. 2006); ***Nicotiana tabacum*(2)**(Ruttink et al. 2006); ***Sesbania rostrata*(1)**(Corich et al. 1998); ***Medicago sativa*(2)**(Kouchi et al. 1999); ***Casuarina glauca*(1)**(Santi et al. 2003); ***Trifolium repens*(3)**(Varkonyi-Gasic and White Derek 2002); ***Lycopersicon esculentum*(1)**(Vleghels et al. 2003); ***Lupinus luteus*(2)**(Podkowinski et al. 2009); ***Pisum sativum*(1)**, ***Vicia sativa*(1)**, ***Cicer arietinum*(2)**, ***Glycine max*(4)**, ***Phaseolus vulgaris*(2)**, ***Vigna radiata*(2)**, ***Macrotyloma uniflorum*(2)**, ***Cajanus cajan*(2)**, ***Abrus precatorius*(2)**, ***Arachis hypogaea*(4)**, ***Aeschynomene evenia*(2)**, ***Nissolia schottii*(2)**, ***Lupinus angustifolius*(4)**, ***Lupinus albus*(3)**, ***Prosopis alba*(2)**, ***Mimosa pudica*(2)**, ***Vachellia collinsii*(2)**, ***Senna tora*(2)**, ***Chamaecrista fasciculata*(2)**, ***Eperua falcata*(1)**, ***Ulmus americana*(1)**, ***Morus alba*(1)**, ***Artocarpus camansi*(2)**, ***Pyrus betulifolia*(1)**, ***Cydonia oblonga*(1)**, ***Rosa chinensis (1*)**, ***Fragaria vesca*(1)**,***Geum urbanum*(2)**, ***Purshia tridentata*(1)**, ***Dryas drummondii*(1)**, ***Begonia fuchsioides*(1)**, ***Cucumis sativus*(1)**, ***Cucumis melo*(1)**, ***Lagenaria siceraria*(1)**, ***Benincasa hispida*(1)**, ***Citrullus lanatus*(1)**, ***Cucurbita pepo*(1)**, ***Luffa acutangular*(1)**, ***Castanea mollissima*(1)**, ***Castanea crenata*(1)**, ***Quercus gilva*(1)**, ***Quercus glauca*(1)**, ***Quercus wislizeni*(1)**, ***Quercus robur*(1)**, ***Quercus mongolica*(1)**, ***Quercus lobata*(1)**, ***Fagus sylvatica*(1)**, ***Fagus crenata*(1)**, ***Casuarina equisetifolia*(1)**, ***Betula pendula*(1)**, ***Betula nana*(1)**, ***Alnus glutinosa*(1)**, ***Corylus heterophylla*(1)**, ***Morella rubra*(1)**, ***Juglans regia*(2)**, ***Juglans californica*(1)**, ***Juglans hindsii*(1)**, ***Juglans macrocarpa*(1)**, ***Juglans nigra*(1)**, ***Juglans sigillata*(2)**, ***Juglans cathayensis*(2)**, ***Juglans mandshurica*(2)**, ***Carya cathayensis*(2)**, ***Carya illinoinensis*(2) and *Pterocarya stenoptera*(2)**(Gultyaev et al. 2023); ***Prunus armeniaca*(1)**, ***Populus tremula*(1)**, ***Euphorbia tirucalli*(1)**, ***Bruguiera gymnorrhiza*(1)**, ***Manihot esculenta*(1)**, ***Eucalyptus gunnii*(1)**, ***Gossypium hirsutum*(1)**, ***Citrus sinensis*(1)**, ***Arabidopsis thaliana*(1)**, ***Citrus unshiu*(1)**, ***Thlaspi caerulescens*(1)**, ***Brassica napus*(1)**, ***Daucus carota*(1)**, ***Helianthus annuus*(1)**,***Senecio aethnensis*(1)**, ***Lactuca sativa*(1)**,** T*araxacum officina*(1)**, ***Solanum tuberosum*(1)**, ***Antirrhinum majus*(1)**, ***Plantago major*(1)**,** and *Hedyotis terminalis*(1)**(Gultyaev and Roussis 2007).
**RTFL/DVL**	***Oryza sativa*(24)**(Wen et al. 2004); ***Brachypodium distachyon*(5)**, ***Sorghum bicolor*(4)**, ***Zea mays*(10) and *Hordeum vulgare*(3)**(Guo et al. 2015).	***Arabidopsis thaliana*(21)**(Wen et al. 2004); ***Gossypium arboretum*(20)**, ***Gossypium raimondii*(19)**, ***Gossypium hirsutum*(39) and *Gossypium barbadense*(39)**(Jiao et al. 2024); ***Medicago truncatula*(17), *Ricinus communis*(8)**, ***Carica papaya*(2)**, ***Glycine max*(40)**, ***Populus trichocarpa*(14)**, ***Vitis vinifera*(2)**, ***Fragaria vesca*(3)**, ***Arabidopsis lyrate*(17)**, ***Thellungiella halophila*(7)**, ***Thellungiella parvula*(5)**, ***Cleome spinosa*(2)**, ***Solanum lycopersicum*(3)**,** and *Aquilegia caerulea*(6)**(Guo et al. 2015)

In general, secretory peptide hormones are firstly produced as preproproteins in plant cells by translations in the ER, and subsequently transported through the Golgi apparatus, and then to outside the cell by secretion, or to the cell membrane. During these processes, post-translational cleavages and modifications occur (Hou et al. 2014; Corpo et al. 2024). This type of peptide hormones are the most common ones identified so far in plants, in which their preproproteins often carry an N-terminal signal sequence that guides their transports through the secretory pathway (exocytosis). Secretory peptide hormones can be further divided to different groups based on their PTMs and Cysteine richness (Cys-rich) (Table 1; Fig. 1) (Tavormina et al. 2015).

Studies on plant peptide hormones with PTMs have underscored their importance in a wide range of biological processes including plant growth and development, and defense responses (Taleski et al. 2018; Fletcher 2020; He et al. 2024). For instance, the hydroxylations and glycosylations of proline residues in CLE peptides, particularly in the conserved CLE motif, are important for their maturation, stability, and receptor interaction (Matsubayashi 2011). PSKs, a family of sulfated pentapeptides, play a critical role in promoting cell proliferation and differentiation (He et al. 2024). A well-characterized example of PTMs for peptide hormones is that the maturation of PSKs involves sulfations on the Tyr residue in their core sequence of YIYTQ, which is essential for their biological activities and binding affinities to receptors. Hydroxyproline-rich systemin (HypSys) peptides are characterized by post-translational hydroxylation on their proline residues, and are implicated in systemic responses to wounding and pathogen attacks (Narváez-Vásquez et al. 2007; Bhattacharya et al. 2013). Other peptide hormones such as CEPs, INFLORESCENCE DEFICIENT IN ABSCISSION (IDA) and IDA-LIKE (IDLs), and PLANT PEPTIDE CONTAINING SULFATED TYROSINE 1 (PSY1), also exhibited critical PTMs during their maturations (Olsson et al. 2019).

In addition to specific PTMs, cysteine (Cys)-rich peptide hormones have the potential to form disulfide bonds between sulfur atoms in two Cys residues, which are often essential for proper folding, stability, biological activities, and resistance to proteolytic degradation. Peptide hormones in this group typically contain between 2 and 16 Cys residues, with notable variations in length and primary sequences (Hou et al. 2014; Tavormina et al. 2015). Many Cys-rich peptide hormones including PLANT DEFENSINs (PDFs), defensin-likes (DEFLs), RALFs, non-specific LIPID TRANSFER PROTEINS (nsLTPs) and KNOTTIN, have been reported to act as modulators in immune responses in plants (Silverstein et al. 2007; De Coninck et al. 2013; Souza et al. 2014; Missaoui et al. 2022; Tang et al. 2022). Cys-rich peptides such as RALFs, S-LOCUS CYSTEINE-RICH PROTEIN/S-LOCUS PROTEIN11 (SCR/SP11), EMBRYO SAC4 (ES4), EMBRYO SURROUNDING FACTOR1 (ESF1) and LUREs are also involved in plant growth and development (Okuda et al. 2009; Takeuchi 2021).

The extracellular delivery of non-secretory peptide hormones, on the other hand, may not follow the classical secretory pathway (Fig. 1). These peptides often lack an N-terminal signal sequence and are localized in various cellular compartments such as the cytoplasm, nuclei, chloroplasts or mitochondria. Some of these peptides go through various unconventional secretory pathways (see the “Maturations of peptide hormones” section) and function at the intercellular space like the SSPs, while some others function within the cell where they are synthesized (Fig. 1). Non-secretory peptides often contain proline (Pro), glycine (Gly), tyrosine (Tyr) or lysine (Lys) residues in their primary sequences. Families of non-secretory peptide hormones include 18-amino acid SYS, 23- to 36-amino acid PEPs, and lysine-rich 11-amino acid GRI peptide, which have been shown to be involved in plant defense responses (Matsubayashi 2014; Tavormina et al. 2015).

### The maturations of peptide hormones

The production of peptide hormones from preproproteins in plants involves several critical steps including the removal of signal peptide and folding, proteolytic processing and PTMs by specific enzymes. Ultimately, these peptides are secreted out of the cell through either the conventional protein secretion (CPS) pathway that involves ERs, Golgi apparatus and endomembrane system, or the unconventional protein secretion (UPS) pathways such as secretory vesicles or granules, secretory multivesicular bodies, vacuoles, and excyst-positive organelles (EXPO) (Fig. 1) (Hou et al. 2014; Corpo et al. 2024).

Peptide hormones produced after PTMs typically consist of less than 20 amino acid residues, and have none or a few Cys residues, and contain PMTs on residues such as Pro and Tyr. The maturation processes of these peptides involve reactions catalyzed by specific enzymes (Table 3). For instance, PTMs executed by Pro hydroxylation and C-terminal processing are required for the activity of the CLE40 peptide. During this process, the CLE40 preproprotein is proteolytic cleaved by subtilisin-like serine proteases (SBTs) SBT1.4, SBT1.7 and SBT4.13, which ultimately release the active peptide (Stührwohldt et al. 2020a). Moreover, the IDA/IDL family peptide hormones that are vital for plant development, especially in organ abscission, are processed by subtilisin-like proteinases (SBT4.12, SBT4.13 and SBT5.2) to produce their mature forms with 14 amino acid residues (Schardon et al. 2016). Essential residues for the maturation of IDA peptides include Pro and Tyr at the position 2 (P2) and P4 positions, respectively, as substitutions of these residues impair the recognition of the IDA precursor (proIDA) peptide by SBT4.13, consequently diminishing its ability to rescue the abscission defects observed in the *ida* mutants (Stenvik et al. 2008).

**Table 3:** Peptide hormones in plants with reported processing proteinases

Peptide hormones	Pre-protein lengths (AA)	Proteases involved in processing	Cleavage sites	Structural components (Count of: Helix-Loop-Sheet)^a^	References
flg22	192	SBT5.2 and SBT1.7	Asn^39^-Ser^40^ and Ala^51^ -Thr ^52^	2–11-8	(Matsui et al. 2024).
CLE40	80	SBT4.13;	Glu^61^ -Arg^62^ and His^73^ -Lys^74^	1–2-0	(Stührwohldt et al. 2020a).
GLV1	147	SBT6.1 and SBT3.8	Arg^95^-Ser^96^and Leu^11^-Gly^120^; Met^131^ -Asp^132^ and Glu^145^ -Lys^146^	4–5-0	(Ghorbani et al. 2016); (Stührwohldt et al. 2020b).
IDA	77	SBT4.12 SBT4.13 and SBT5.2	Lys^55^ -Gly^56^ and Asn^69^ -Ser^70^	2–3-0	(Schardon et al. 2016).
AtPSK1	87	SBT3.8	Asp^76^-Tyr^77^ and Gln^81^ -Asp^82^	2–3-0	(Stührwohldt et al. 2021).
SlPSK1	90	SlPhyt2	Asp^80^-Tyr^81^ and Gln^85^ -His^86^	3–4-0	(Reichardt et al. 2020).
AtPSK4	79	SBT1.1	Asp^70^ -Tyr^71^ and Gln^75^ - Asn^76^	2–3-0	(Srivastava et al. 2008).
EPF2	120	CRSP	Asp^75^ -Cys^76^	2–6-3	(Engineer et al. 2014).
RALF23	138	AtS1P	Asn^84^ -Arg^85^ and Leu^88^ -Ala^89^	4–5-0	(Srivastava et al. 2009).
Systemin	200	SlPhyt1, SlPhyt 2 and LapA	Asp^177^**-**Leu^178^; Arg^188^**-**Asp^189^; Asp^196^**-**Asn^197^	0–1-0	(Beloshistov et al. 2018); (Gu and Walling 2000)
PEP1	92	MC4 to MC9	Arg^63^ -Aal^64^	1–2-0	(Hander et al. 2019); (Shen et al. 2019)
SCOOP12	78	SBT3.5	Gly^41^ -Arg^42^ and Met^45^ -Gly^46^	1–2-0	(Yang et al. 2023).
TWS1	81	SBT1.8 and ALE1	Glu^31^ -Asp^32^ and His^54^ -Gly^55^	1–2-0	(Royek et al. 2022).
CIF4	102	SBT5.4	Gly^66^ -Asp^67^ and His^89^ -Gly^90^	3–4-0	(Truskina et al. 2022).

^a^Based on structure prediction via AlphaFold (https://alphafold.ebi.ac.uk/)

Proteolytic processing of peptide hormones could be important regulatory point in signal transduction. Proteolytic cleavage of the PEP1 preproprotein by Ca^2+^-dependent type-II metacaspases (MCs) is required for the maturation of PEP1 peptides. Upon wounding, MC4 was activated by binding to high levels of Ca^2+^, which is necessary and sufficient for the PEP1 maturation (Hander et al. 2019). Another study showed that the processing of PEP1 peptide was blocked by displacing the catalytic Cys residue in MC4, or the conserved Arg residue preceding the PEP1 domain in the precursor protein (Shen et al. 2019).

PSKs are a class of sulfated peptides crucial for plant growth and development. PSKs are produced firstly by translation as 80–110 amino acid preproprotein precursors, and processed into sulfated mature peptides with five amino acid residues. The maturation of PSK peptides involves Tyr sulfation executed by tyrosylprotein sulfotransferase (TPST) in the cis-Golgi, and proteolytic cleaved by SBT1.1/SBT3.8 in the apoplast (Srivastava et al. 2008; Stührwohldt et al. 2021). The *Arabidopsis thaliana* TPST is responsible for catalyzing the transfer of sulfate from 3′-phosphoadenosine 5′-phosphosulfate to Tyr residues within the acidic motifs of the PSK peptides (Komori et al. 2009). In addition to PSKs, TPST is also involved in Tyr sulfation of multiple peptide families including PSYs (Komori et al. 2009), RGFs (Zhong et al. 2020) and CIFs (Okuda et al. 2020). Results from genetic analyses and receptor-ligand interaction assays indicated that the TPST catalyzed Tyr sulfation is critical for receptor-binding and peptide signaling (Okuda et al. 2020; Zhong et al. 2020; Kaufmann et al. 2021).

Pro hydroxylation followed by hydroxyproline arabinosylation is a pattern of PTMs observed in a number of peptide families including CLEs, CEPs, RGFs, PSYs, Sys (Stührwohldt and Schaller 2019), and Ser-rich endogenous peptides (SCOOPs) (Guillou et al. 2022a). In most cases, glycolsylation of these peptides have a positive effect on receptor binding and biological activities (Okamoto et al. 2013; Imin et al. 2018). In CEP1-D1 of *Medicago truncatula*, however, tri-arabinosylation of the peptide showed negative effect on its function in promoting nodule formation and inhibiting lateral root development (Patel et al. 2018). For several other peptides such as TRACHEARY ELEMENT DIFFERENTIATION INHIBITORY FACTOR (TDIF) (CLE41/44) of *Arabidopsis thaliana*, Pro hydroxylation seemed to be not required for activities (Ito et al. 2006; Zhang et al. 2016a). Interestingly, for *Arabidopsis thaliana* CLE40 peptides, hydroxylation and arabinosylation of the Pro at P4 did not change its activity in root growth inhibition. Instead, the hydroxylation showed a significant impact on cleavage site selection during proteolytic processing by SBT subtilases, resulting in differentially processing of CLE40 preproproteins (Stührwohldt et al. 2020a). Pro hydroxylation and hydroxyproline arabinosylation are believed to be catalyzed by prolyl-4-hydroxylases (P4Hs) and arabinofuranosyltranferases (ArafTs) (Petersen et al. 2021), however, so far no specific enzymes have been identified yet.

### Structural features of peptide hormones

The structural features of peptide hormones vary widely, and are crucial for their biological functions. Structure features include sizes and compositions of amino acid residues, and three-dimensional structures of peptides. These may contribute to stability, bioactivities, and specificity of peptide hormones, and may affect their interactions with other molecules including receptor kinases (Table 3).

Peptides produced from the C-terminal conserved domains of CLE-related preproproteins consist of a 12- to 13-amino acid motif (Cock and McCormick 2001). This motif often has 1–3 Pro residues that are conserved, and typically hydroxylated to form hydroxyproline, a modification important for their biological activities. For example, the biological activity of the CLV3 peptide hormone is significantly enhanced by the modification with a tri-arabinoside to a hydroxyproline, which causes a structural rearrangement in the peptide backbone, and maximizes its functionality (Miyawaki et al. 2013; Shinohara and Matsubayashi 2013). The molecular mechanism underlying these modifications however, remains largely unknown. CEPs are another family of putative peptide hormones with PTMs in a conserved C-terminal domain. *Arabidopsis thaliana* CEP1 (AtCEP1), in its mature form of 15 amino acid residues, undergoes modifications at two Pro residues, leading to the formation of a β-turn-like conformation that is critical for receptor binding (Bobay et al. 2013). Similar structural change has also been hypothesized for *M. truncatula* CEP1 (MtCEP1) and *Meloidogyne hapla* CEP1 (MhCEP1). cysteine-rich proteins (CRPs) such as SCR/SP11 exhibit more complex structural features. SCR/SP11 includes an L1 loop, an α-helix, and a β-sheet, with the L1 loop and α-helix playing crucial roles in receptor binding (Mishima et al. 2003). Lipid transfer proteins (LTPs) have four α-helices and three loops connected by four disulfide bridges formed from eight conserved Cys residues, contributing to their stabilities and functionalities in lipid transfer (Hou et al. 2014). Stomagen, a peptide hormone in the EPF family, is featured with a loop, two antiparallel β-strands and a scaffold stabilized by three disulfide bonds (Ohki et al. 2011). Despite rapid advances in bioinformatics and mass spectrometry tools, only a limited number of peptide hormones have been characterized for the molecular properties of their mature forms due to their low abundances in plants.

## The roles of well-characterized peptide families in plants

### Systemin peptides

Many plants respond to insect attack and wounding by activating the expression of genes involved in herbivore deterrence, wound healing, and other defense-related processes. A fascinating feature of these inducible defenses is their occurrence both locally at the site of wounding and systemically in remote undamaged leaves. It has been proposed more than 50 years ago that specific mobile signals, generated at the wound site, travel throughout the plant body and activate expressions of defense-related genes in systemic responding leaves (Green and Ryan 1972). Wound-inducible defensive proteinase inhibitors of tomato provide an attractive model system to investigate the mechanism of systemic defense responses. Among the well-established intercellular signals promoting systemic defense responses are systemin, the first bioactive peptide isolated from plants, and the wound hormone jasmonate (Farmer and Ryan 1990; Pearce et al. 1991). Systemin is an 18-amino-acid immunomodulatory peptide cleaved from a precursor protein called prosystemin (Pearce et al. 1991). Transgenic tomato plants expressing an antisense *PROSYSTEMIN* (*PS*) gene lacked systemic defense responses (McGurl et al. 1992). Conversely, transgenic tomato plants (*35 S::PS*) that overexpress *PS* constitutively expressed systemic defense responses without wounding and were more resistant to insects (McGurl et al. 1994). In addition, jasmonate mutants can suppress the constitutive wound signaling phenotype of *35 S::PS* plants (Li et al. 2003; Yan et al. 2013). These genetic studies, together with a wealth of other evidence, led to a model in which systemin functions upstream of jasmonate and that these two signals act through a common signaling pathway to regulate systemic defense responses (Ryan 2000; Schilmiller and Howe 2005; Sun et al. 2011).

**Fig. 2:**
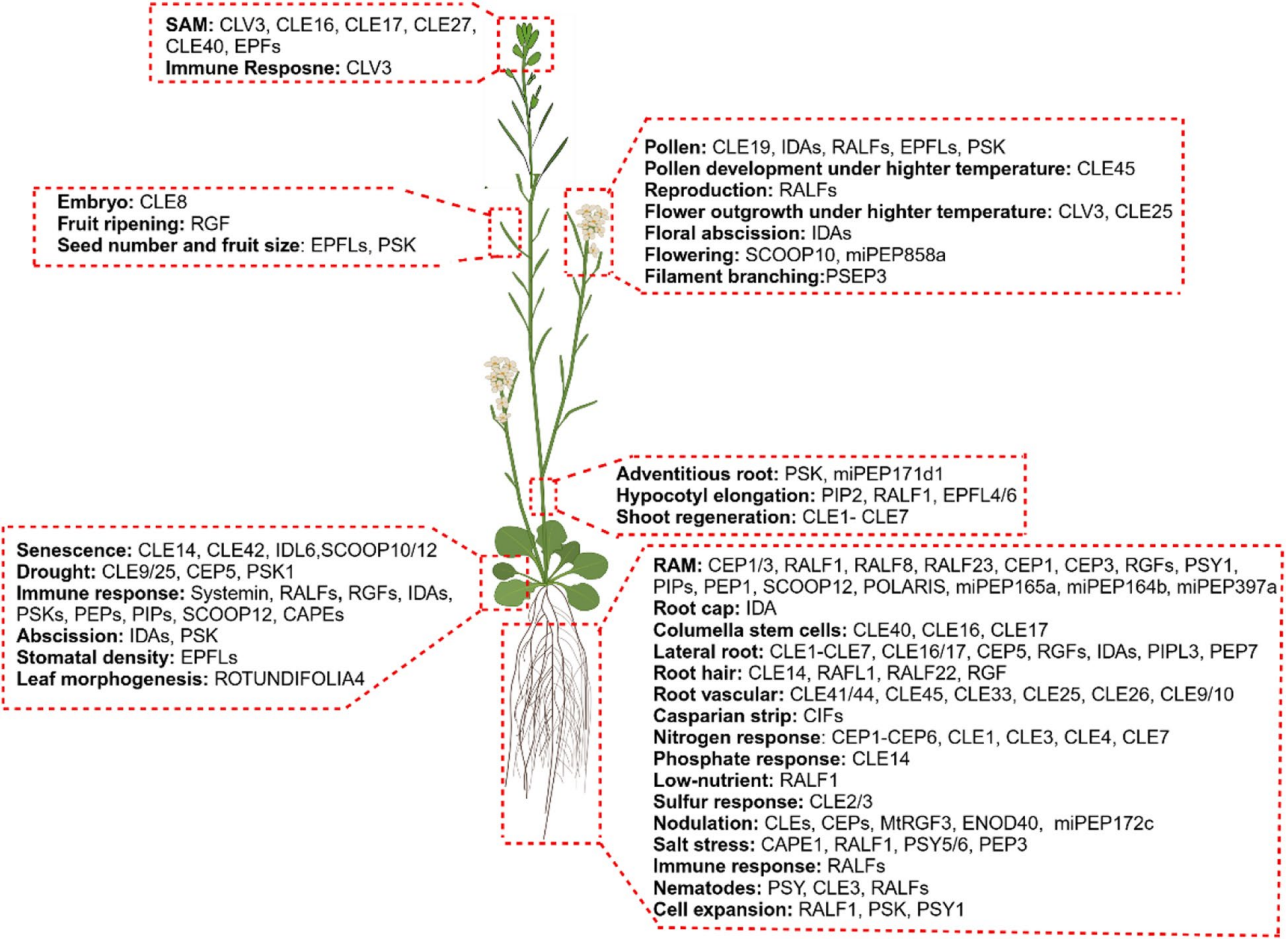
Functional roles and diversity of plant peptides Plant peptides play diverse biological roles across various tissues, contributing to growth, development, and responses to both abiotic and biotic stresses

Upon its discovery, systemin was initially considered to be the long sought-after systemic wound signal (Pearce et al. 1991; Ryan 2000). However, grafting experiments with tomato mutants defective in jasmonate and/or systemin signaling provided evidence that systemin acts locally at the site of wounding, where it amplifies jasmonate production to threshold levels that are required for the activation of systemic defense responses (Li et al. 2002, 2003; Ryan and Moura 2002; Sun et al. 2011). This proposed mode of action of systemin in the amplification of systemic immunity shares similarities to metazoan cytokines (Gust et al. 2017). From this perspective, systemin and other related host-derived, damage-associated molecular patterns (DAMPs) may be termed immunomodulatory phytocytokines (Gust et al. 2017) (Fig. 2).

### Clavata3/Embryo Surroundingr Region (CLE) peptides

The CLE family peptides are widely known for their critical functions in modulating stem cell homeostasis across various meristematic tissues, including the shoot apical meristem (SAM), the root apical meristem (RAM), and the procambium (vascular meristem) (Song et al. 2022b; Selby and Jones 2023) (Fig. 2). In the SAM, *clv3* mutants exhibited an increased number of stem cells and consequently an enlarged SAM (Clark et al. 1995; Fletcher et al. 1999). CLV3 signaling, which is perceived by several receptors including CLV1 and its homologs BARELY ANY MERISTEMs (BAMs), CLV2-CORYNE (CRN), RECEPTOR-LIKE KINASE 2 (RPK2), and co-receptors CLV3 INSENSITIVE KINASES (CIKs)/CLE-RESISTANT RECEPTOR KINASE (CLERK), limits the expression of the homeodomain transcription factor gene *WUSCHEL* (*WUS*). Conversely, WUS promotes the expression of *CLV3*, thereby forming a dynamic negative feedback loop that regulates stem cell homeostasis in the SAM (Clark et al. 1997; Schoof et al. 2000; Brand et al. 2000; Müller et al. 2008; DeYoung and Clark 2008; Kinoshita et al. 2010; Nimchuk et al. 2015; Anne et al. 2018; Hu et al. 2018; Ren et al. 2019). Through domain deletion analyses, it has been shown that the 14-amino acid CLE motif of CLV3 is essential and sufficient to execute its role in the stem cell maintenance in vitro (Fiers et al. 2006), revealing that the CLE motif functions as the peptide hormone for stem cell maintenance (Fiers et al. 2007). Further, the glycine residue within the CLE motif of CLE proteins is critical for their functions, as showed by amino acid substitution experiments (Song et al. 2013). Substituting the Gly with Thr led to dominant- negative phenotypes when substitution constructs were expressed under their endogenous regulatory elements and introduced to the wild type *Arabidopsis thaliana* (Song et al. 2013). Other than CLV3, other CLE peptides including CLE16, CLE17, and CLE27 are also involved in stem cell homeostasis in vegetative and/or reproductive SAMs (Jun et al. 2010). Moreover, although single and double mutants of *CLE16*, *CLE17*, and *CLE27* did not exhibit any detectable phenotypes in the SAM (Gregory et al. 2018), these genes redundantly contribute to stem cell maintenance of the SAM (Dao et al. 2022). *CLE40* is expressed in differentiating cells in a complementary pattern as *CLV3* in the SAM, where CLE40 elevates the levels of *WUS* via BAM1, thus increasing the stem cell number (Schlegel et al. 2021). Therefore, the stem cell homeostasis in the SAM seems to be coordinated by two antagonistic CLE peptide signaling pathways: the CLV3-CLV1 pathway in restricting the stem cell number and the CLE40-BAM1 pathway in regulating the cell differentiation (Schlegel et al. 2021). Intriguingly, active compensation operates in CLE ligands, to control the SAM stem cell number in tomato. *SlCLE9*, the closest paralog of *SlCLV3*, is upregulated upon the loss of *SlCLV3*, buffering stem cell homeostasis in tomato (Rodriguez-Leal et al. 2019).

Exogenous application of synthetic CLE peptides and/or overexpression of corresponding *CLE* genes have been shown to change root architecture, leading to primary root growth termination and/or defects in lateral root development (Fiers et al. 2004, 2005; Strabala et al. 2006; Kinoshita et al. 2007; Wang and Fiers 2010; Betsuyaku et al. 2011). Application of chemically synthetic CLE peptides (corresponding CLE motifs) triggered the termination of the RAM and led to short-root phenotypes (Fiers et al. 2005; Kinoshita et al. 2007; Strabala et al. 2006). Thus, these peptides are called root-active CLE peptides. The *clv2*, *crn* or *rpk2* mutants are insensitive to these root-active CLE peptides, indicating that CLV2, CRN, and RPK2 are needed for recognizing these root-active CLE peptides and regulating the proximal root meristem (Fiers et al. 2005; Kinoshita et al. 2007; Miwa et al. 2008; Müller et al. 2008; Stahl et al. 2009; Hazak et al. 2017). In the RAM, *cle40* mutant exhibited increased number of columella stem cells (CSC), whereas application of CLE40 peptide reduced the number of CSC (Stahl et al. 2009). The receptors, including ARABIDOPSIS CRINKLY4 (ACR4), CLV1, RPK2, and CIKs/CLERKs, are required for CLE-mediated root stem cell homeostasis executed by regulating the expression of *WUSCHEL*-*RELATED HOMEOBOX5* (*WOX5*) (Stahl et al. 2009, 2013; Kinoshita et al. 2010; Ren et al. 2019; Zhu et al. 2021b). CLE16/CLE17 peptides are also involved in CSC differentiation through the CLV1 and ACR4 receptors in the distal root meristem (Zhang et al. 2022b). In addition, CLE16/CLE17 peptides promote lateral root emergence (Zhang et al. 2022b), while CLE1-CLE7 suppress lateral root emergence and elongation (Araya et al. 2014; Nakagami et al. 2023). Consistently, the septuple mutant of *cle1*-*cle7* correspondingly exhibited longer lateral roots and increased lateral root density (Nakagami et al. 2023). Regarding root hair development, CLE14 peptide application induced excessive root hair formation and root hair growth by reducing the expression of *GLABRA 2* (*GL2*) under normal growth conditions (Hayashi et al. 2018).

The proliferation and differentiation of (pro-)cambium stem cells in the vascular system are also redundantly regulated by CLE peptides (Qiang et al. 2013; Fukuda and Hardtke 2020). Notably, phloem-derived CLE peptides TDIF/CLE41/CLE44 promote proliferation and inhibit differentiation of vascular cambium cells by acting through the TDIF RECEPTOR (TDR)/PHLOEM INTERCALATED WITH XYLEM (PXY) receptor, and regulating *WOX4* and *WOX14* expression (Hirakawa et al. 2008, 2010; Whitford et al. 2008; Etchells and Turner 2010). However, a different set of phloem-derived CLE peptides, including CLE25, CLE26, CLE33, and CLE45, function to inhibit phloem formation and maintain the proximal root meristem (Depuydt et al. 2013; Ren et al. 2019; Carbonnel et al. 2023). Several studies revealed that these phloem-formation inhibitory CLE peptides are perceived through paralleled receptors, including CLV2/CRN, BAM1/BAM3, and CLERK/CIK (Depuydt et al. 2013; Ren et al. 2019; Hu et al. 2022). Regarding the xylem formation, overexpression of *Brassica napus CLE19* led to formations of disconnected xylem and vascular islands in flower buds (Fiers et al. 2004). It has been found that CLE9/CLE10 inhibited protoxylem formation by repressing the expression of *ARR5* and *ARR6* in *Arabidopsis thaliana* roots (Kondo et al. 2011).

Given the prominent roles that CLE peptides play in determining stem cell fate in different types of meristems, it is envisaged that some peptides may also be involved in plant regeneration, which requires reprogramming of differentiated cells to stem cells and establishing nascent meristems (Wang et al. 2022c). Indeed, many siganling peptide-encoding genes, including multiple *CLE* genes are expressed in callus that is a mass of pluripotent cells capable of giving rise to shoots or roots (Wang et al. 2022c). CLE1-CLE7 peptides can effectively suppress *de novo* shoot regeneration, whereas the septuple mutant of *cle1-cle7* displays an enhanced regeneration capacity (Kang et al. 2022). It is indicated that these functionally redundant CLE1-CLE7 peptides act through CLV1/BAM1 receptors to repress *WUS* expression, thereby, modulating shoot-regeneration (Kang et al. 2022).

CLE peptides also play important roles in plant reproduction. *CLE8* is highly expressed in young embryos and endosperms, and the *cle8* mutant produces smaller and defective seeds/embryos (Fiume and Fletcher 2012). It is plausible that CLE8 regulates seed size and growth through promoting *WOX8* expression (Fiume and Fletcher 2012). *CLE19* expression is confined to the embryo, specifically in epidermial cells in cotyledon and hypocotyl of the embryo. Transgenic plants expressing an antagonistic *CLE19* construct exhibits seed abortion phenotypes, showing defective cotyledon establishment in embryos and delayed nuclear proliferation and cellularization in endosperms (Xu et al. 2015). Furthermore, it has been showed that, in pollen development, the CLE19 signal is perceived by PXY-LIKE1 (PXL1) to coordinate the expression of tapetal regulators, thereby maintaining normal pollen development in *Arabidopsis thaliana* (Wang et al. 2017b; Yu et al. 2023c).

Leaf senescence can be regulated by various internal and environmental factors, including peptide hormones (Han et al. 2022). Loss-of-function mutation in *CLE14* resulted in precocious leaf senescence. In contrast, plants overexpressing *CLE14* gene, or treated with synthetic CLE14 peptides, exhibits delayed leaf senescence (Han et al. 2022; Zhang et al. 2022h). *CLE14* overexpression transcriptionally activated *JUB1*, a negative regulator of senescence, through reactive oxygen species (ROS) scavenging to regulate age-dependent and stress-induced leaf senescence (Zhang et al. 2022h). Similarly, *cle42* mutant exhibits earlier leaf senescence phenotypes, whereas *CLE42* overexpression resulted in delayed senescence (Zhang et al. 2022g). It has been proposed that CLE42 delays leaf senescence through antagonizing the ethylene pathway by controlling ethylene biosynthesis (Zhang et al. 2022g). It appears that CLE14 and CLE42 inhibit leaf senescence via distinct cellular regulatory networks.

Furthermore, CLE peptides also play significant roles in plant adaptations such as biotic and abiotic stress responses and nutrient sensing (Wang et al. 2016; Zhang et al. 2022f; Xie et al. 2022a; Bashyal et al. 2024). The expression of a range of *CLE* genes, including *CLV3*, is perturbed by phytohormones and environmental stimuli (Wang et al. 2016; Yang et al. 2017). CLV3, the key regulator of stem cell homeostasis in the SAM, is likely to be recognized by flagellin receptor kinase FLAGELLIN SENSITIVE 2 (FLS2) to trigger immune signaling and enhance pathogen resistance in the SAM (Lee et al. 2011). Moreover, the CLV3 peptide may act thorugh CLV1 and CLV2 receptors to modulate the defense response in plants against *Ralstonia solanacearum* through the miR169/NUCLEAR TRANSCRIPTION FACTOR Y SUBUNIT A (NF-YA) module (Hanemian et al. 2016). CLE9 and/or CLE25 peptides accelerate stomatal closure, resulting in enhanced drought tolerance, whereas *cle9* and/or *cle25* loss-of-function mutants exhibit elevated sensitivities to drought stress (Takahashi et al. 2018; Zhang et al. 2019a). Further analysis shows that CLE9 and CLE25 trigger different but overlapped signaling pathways to promote stomatal movement (Takahashi et al. 2018; Zhang et al. 2019a). Treatments with CLE45 peptide, which binds to SKM1/SKM2 receptors, prolongs pollen tube growth at high temperatures without affecting pollen germination (Endo et al. 2013). CLV3 and CLE25 peptides are also implicated in plant adaption to temperature stress, as evidenced that both of these two CLE peptides regulate flower outgrowth under varying temperatures (Jones et al. 2021; John et al. 2023).

Under the low nitrogen, phosphate, or sulfur conditions, the expressions of a number of *CLE* genes have been shown to be differentially regulated in roots, resulting in impaired root meristem differentiation and lateral root formation (Araya et al. 2014; Gutiérrez-Alanís et al. 2017; Dong et al. 2019). The expressions of *CLE1*, *CLE3*, *CLE4* and *CLE7* are elevated in roots under the low nitrogen condition, and under such conditions CLE3 is perceived by the phloem-localized CLV1 to inhibit lateral root development (Araya et al. 2014). Under the phosphate starvation, *CLE14* transcription is up-regulated, and the CLE14 signal is perceived by CLV2 and PEPR2 to trigger root meristem termination through repressing the expressions of *SHORT ROOT* (*SHR*), *SCARECROW* (*SCR*) and *WOX5* (Gutiérrez-Alanís et al. 2017). Sulfur deficiency reduces *CLE2* and *CLE3* expression levels, resulting in decreased lateral root density, which is diminished in *clv1* mutants, suggesting that the CLE-CLV1 module controls macronutrient utilization through affecting root architecture (Dong et al. 2019).

CLE genes have been identified throughout the land plant lineage (Oelkers et al. 2008; Goad et al. 2017), as well as in plant-parasitic nematodes (Wang et al. 2005). The peptide ligand CLV3 and its homologs/orthologs coordinate stem cell homeostasis through a negative feedback loop which is highly conserved across the plant kingdom (Somssich et al. 2016; Hirakawa and Sawa 2019). In liverwort *Marchantia polymorpha*, the MpCLE1 (TDIF-type)-MpTDR module serves as a negative regulator of stem cell proliferation, while the MpCLE2 (CLV3-type)-MpCLV1 module functions as a positive regulator of stem cell activity in the apical notch (Hirakawa et al. 2019, 2020). FLORAL ORGAN NUMBER2 (FON2)/CLV3 and its homolog FON2-LIKE CLE PROTEIN1 (FCP1) are sub-functionalized in controlling stem cell homestasis of rice shoot meristems (Suzaki et al. 2006, 2008, 2009; Chou et al. 2006). Similarly, mutations in maize *ZmCLE7* (ortholog of *FON2*) and *ZmFCP1* (ortholog of *FCP1*) led to stem cell over-proliferation, resulting in meristem enlargement and excess organs (Je et al. 2016, 2018; Rodriguez-Leal et al. 2019). The *fasciated* (*fas*) mutant in tomato, showing increased locule numbers in anther, is caused by a mutation in a *CLV3* orthologous gene (Rodriguez-Leal et al. 2019). In some legume plants, CLE peptides are involved in auto-regulation of nodulation (AON). The AON signaling induces productions of CLE peptides in *Medicago truncatula* (Mortier et al. 2010; Moreau et al. 2021; Mens et al. 2021) and *Lotus japonicus* (Okamoto et al. 2009, 2013; Nishida et al. 2018). These CLE peptides are recognized by SUPER NUMERIC NODULES (SUNN) from *M. truncatula* and HYPERNODULATON AND ABERRANT ROOT 1 (HAR1) from *L. japonicus* to reduce nodule numbers (Mortier et al. 2010; Okamoto et al. 2013; Mens et al. 2021).

### C-Terminally Encoded Peptide (CEP) peptides

CEP peptides have been discovered in many plant species (Delay et al. 2013; Roberts et al. 2013; Ogilvie et al. 2014) (Fig. 2), conferring critical roles in primary and lateral root development, nodulation formation, nitrogen uptake and transport, and stress acclimatization (Roberts et al. 2013; Taleski et al. 2018, 2024). The application of CEP peptides and ectopic expression of *CEP* genes significantly primary repress root elongation in a CEPR receptor-dependent manner (Ohyama et al. 2008; Delay et al. 2013; Roberts et al. 2016). Furthermore, it has also been observed that CEP peptide application or *CEP* gene over-expression triggers a reduction of total lateral root number and density (Delay et al. 2013; Roberts et al. 2016). In contrast to *CEP* overexpressors, *cep* mutants exhibited longer primary roots, and longer and increased numbers and densities of lateral roots (Delay et al. 2013; Roberts et al. 2016; Huang et al. 2023) Similarly, MtCEP1 is found to be involved in the inhibition of lateral root formation in *M.truncatula* (Delay et al. 2013; Imin et al. 2013; Mohd-Radzman et al. 2015, 2016). In addition, sucrose up-regulated expressions of many *CEP* genes, consequently resulted in inhibition of lateral root development (Chapman et al. 2019). In *Arabidopsis thaliana* and *Medicago truncatula*, CEP peptides cause shallower root systems (Chapman et al. 2020, 2024), while in *Brassica rapa* and *Cucumis sativus*some CEP peptides exert antagonistic effect on promoting primary root elongation (Liu et al. 2021d; Qiu et al. 2022).

CEP peptides also function as systemic signals to ensure optimized nitrogen uptake, translocation, and nodulation (Okamoto et al. 2016; Xie et al. 2022a). Under the nitrogen starvation conditions, the transcriptional levels of *CEP* genes are elevated in roots (Chu et al. 2021). Subsequently, root-derived CEP peptides are transported to the leaves, where they are perceived by CEPR receptors. CEP-CEPR pair then up-regulates the expression of nitrate transporters, thus promoting nitrogen uptake and transport (Tabata et al. 2014; Ohkubo et al. 2017, 2021; Ota et al. 2020). CEP-mediated nitrogen uptake has also been observed in rice (Sui et al. 2016), apple (Yu et al. 2019b) *M.* *truncatula* (Bourion et al. 2014; Luo et al. 2023). In addition to nitrogen uptake, CEP peptides are positive players in nodule formation. In response to nitrogen starvation, *CEP* expression is up-regulated and CEP peptides bind to the COMPACT ROOT ARCHITECTURE 2 (CRA2) receptor to promote nodule number in *M. truncatula* through acting on the ethylene signaling pathway (Imin et al. 2013; Huault et al. 2014; Mohd-Radzman et al. 2016; Laffont et al. 2020; Luo et al. 2022). CRISPR/Cas9-generated triple and quintuple *cep* mutants displayed developmental defects in lateral root formation and nodulation (Zhu et al. 2021a), and application of MtCEP peptides resulted in an increased nodule formation (Laffont et al. 2020; Zhu et al. 2021a; Ivanovici et al. 2023).

*CEP* expression is dynamically perturbed by abiotic stresses such as drought, salinity and pathogens in roots and shoots (Delay et al. 2013; Smith et al. 2020), indicating the important role of CEP peptides as stress mediators. *CEP3* expression is greatly induced by salinity, and *cep3* mutants displayed longer primary roots under conditions of increased salinity, nitrogen limitation, acidity and osmotic stress (Delay et al. 2013). Treatments with CEP peptides compromised the effects of salt stress on root growth in both cucumber and tomato (Liu et al. 2022a; Shen et al. 2023b). Under N-limited conditions, *CEP4* was significantly up-regulated to confer plant immunity (Rzemieniewski et al. 2022). *CEP5* expression is induced by osmotic stress, and plant overexpressing *CEP5* showed enhanced tolerance to both drought and osmotic stresses by interfering with auxin signaling through either CEPR-dependent or -independent mechanism (Smith et al. 2020). Similarly, applications of SlCEP10 and SlCEP11b peptides enhanced tolerance to drought stresses in tomato (Xu et al. 2023b).

### Rapid Alkalinization Factor (RALF) peptides

RALF peptides play diverse roles in plant development and environmental adaptations (Blackburn et al. 2020; Wang et al. 2022) (Fig. 2). RALF peptides are initially known to trigger cellular alkalization and inhibit root elongation (Pearce et al. 2001b; Du et al. 2016). Treatments with RALF peptides or over-expressions of *RALF* genes often inhibit growth of seedlings and/or roots in *Arabidopsis thaliana* (Abarca et al. 2021). Specifically, *RALF1*, *RALF8* and *RALF23* over-expression lead to the formation of bushy, semi-dwarf plants with small leaves and short roots in a FERONIA (FRE)-dependent manner (Srivastava et al. 2009; Atkinson et al. 2013; Bergonci et al. 2014). In contrast, the *ralf1* knockout mutant shows longer roots and hypocotyls, increased lateral root number, and larger root cells (Bergonci et al. 2014). Additionally, treatments with RALF1 peptide stimulats root hair elongation (Zhu et al. 2020). It seems that the RALF-FER signaling regulates ROS levels and interacts with ABA to regulate the growth of roots and the elongation of root hairs (Duan et al. 2010; Chen et al. 2016). Upon perceiving by FER, RALF1 signaling induces alkalinization of the extracellular matrix through phosphorylating H^+^-ADENOSINE TRIPHOSPHATASE 2 (AHA2) at the plasma membrane and inhibiting proton transport (Haruta et al. 2014). This leads to an increase in external pH, which subsequently impedes the elongation of primary roots and root hairs. It has also been reported that the root growth inhibition mediated by the RALF-FER signaling module requires auxin biosynthetic and auxin signaling pathways (Li et al. 2022b). Ca^2+^ signaling is likely to be a prerequisite for RALF to activate H^+^ transfer and to inhibit root cell expansion. RALF1 increases cytoplasmic C a^2+^ concentrations (Haruta et al. 2008), and blocking of the Ca^2+^ channel prevents extracellular matrix alkalization triggered by RALF treatments (Gjetting et al. 2020).

RALFs are well-studied for their roles at different stages of plant reproduction. On the pollen-stigma surface, RALF23 and RALF33, perceived by the receptor complex FER/ANJEA (ANJ)/LORELEI-LIKE-GPI-ANCHORED PROTEINS (LLGs), inhibit pollen hydration by inducing ROS production through activation of a downstream GTPase pathway (Liu et al. 2021a). When pollen tubes approach ovules, the ovule-expressed RALF34 peptide competes with pollen-expressed RALF4 and RALF19 to bind to the BUDDHA’S PAPER SEAL 1/2-ANXUR 1/2 (BUPS1/2-ANX1/2) receptor complex, facilitating pollen tube apical rupture and sperm cell discharge (Ge et al. 2017). Five pollen tube-produced RALFs, RALF6/7/16/36/37, collectively establish the polytubey block and maintain the double fertilization by binding to the FER/ANJ/HERCULES RECEPTOR KINASE 1 (HERK1) receptor complex (Zhong et al. 2022). Furthermore, stigma-expressed RALF (sRALF) peptides block the penetration of undesired pollen tubes, whereas pollen-expressed RALF (pRALF) peptides counteracted sRALF peptides, enabling successful pollen tube penetration (Lan et al. 2023). RALFs also play crucial roles in crop reproduction. *Pyrus bretschneideri* RALF 2 (PbrRALF2) has been shown to inhibit pollen tube growth by producing excessive ROS (Kou et al. 2021). Similarly, *Solanum lycopersicum* RALF (*Sl*PRALF) specifically inhibited the elongation of pollen tubes (Covey et al. 2010). Silence of *Sc*RALF3 in *Solanum chacoense* led to impaired pollen development (Chevalier et al. 2013; Mazin and Matton 2020).

RALF peptides are also important for abiotic and biotic stress responses, and hormone signaling (Blackburn et al. 2020; Xie et al. 2022a). *RALF1* overexpression transgenic lines exhibit relative resistance to salt stress (Zhao et al. 2018; Feng et al. 2018). Furthermore, the application of RALF1 peptide under salt conditions enhances salt sensitivity (Yu and Assmann 2018). Once perceived by FER, RALF1 peptide inhibits the activities of AHA2 and Na^+^/K^+^ transporters to regulate salt sensitivity (Zhao et al. 2018, 2020; Feng et al. 2018). Additionally, RALF signaling is shown to interact with ABA signaling for abiotic stress responses and possibly stomatal immunity (Yu et al. 2012; Chen et al. 2016). In response to low-nutrient conditions, RALF1 peptide activates the target of rapamycin (TOR) signaling via enhancing FER-TOR interactions (Song et al. 2022a). In moss *Physcomitrium patens*, loss-of-function of *PpRALF2* and *PpRALF3* enhanced resistance to bacterial and fungal pathogens (Mamaeva et al. 2023).

Notably, RALF1, RALF23, and RALF33 peptides have been shown to play crucial roles in modulating the formation of receptor complexes to inhibit immunity responses (Stegmann et al. 2017; Chen et al. 2023). In contrast, RALF17 is shown to be able to induce ROS production and thus induce resistance to *Pseudomonas syringae* pv tomato DC3000 (Stegmann et al. 2017). In principle, RALFs activate a receptor complex containing EF-TU RECEPTOR (EFR)/ FLS2 and FER as a scaffold and trigger downstream immune responses (Stegmann et al. 2017). Furthermore, it is shown that RALF23 acts through FER to elevate jasmonic acid signaling, thus negatively contributing to plant immunity (Guo et al. 2018). Additionally, Ca^2+^ waves, MITOGEN-ACTIVATED PROTEIN KINASE (MAPK) cascades, and ROS bursts are also required for RALF-mediated plant immunity. Under phosphate (Pi)-deprived conditions, RALF signaling mediated by the PHOSPHATE STARVATION RESPONSE 1 (PHR1) is recruited to suppress plant immunity and modulate the rhizosphere microbiome composition (Tang et al. 2022). The RALF23-FER signaling module restricts *Pseudomonas* within the rhizosphere through ROS generation (Song et al. 2021b).

Intriguingly, nematodes and fungi can produce RALF mimics to suppress immune responses and increase disease susceptibility through hijacking the endogenous *planta* FER signaling. Root-knot nematodes *Meloidogyne incognita* have been shown to produce MiRALF1 and MiRALF3 peptides, which are perceived by the host FER receptor to suppress host immune responses and thus enhance parasitic virulence (Plant et al. 2020). F-RALF produced from the fungus *Fusarium* assists the pathogen colonization and increased its virulence in tomato (Masachis et al. 2016). *Colletotrichum tofieldiae* is shown to secrete a RALF-like elicitor, CtRALF, which directly binds to FER to facilitate microbial symbiosis (Liao et al. 2023).

### Root Meristem Growth Factor (RGF)/Golven (GLV)/Cle Like (CLEL) peptides

The family of RGF/GLV/CLEL peptide belongs to the sulfated peptide group and contains 11 members in the *Arabidopsis thaliana* genome (Matsuzaki et al. 2010; Meng et al. 2012; Whitford et al. 2012; Shinohara 2021). RGF peptides are usually perceived by the RGF1 INSENSITIVE (RGI)/ RGF RECEPTOR (RGFR) LRR-RLK receptors. *Arabidopsis thaliana RGF* genes exhibit diverse expression patterns across various organs and tissues, mainly in the root meristem and leaves (Fernandez et al. 2013a). Principally, RGF peptides promote root meristem formation across plant species (Fang et al. 2021) (Fig. 2). Although single mutants of *rgf1*, *rgf2* or *rgf3* do not exhibit root defects, the *rgf1 rgf2 rgf3* triple mutant presents a short-root phenotype which could be restored by RGF1 peptide application (Matsuzaki et al. 2010). Overexpression of various *RGF* genes increases RAM size, inhibits lateral root initiation, and triggers aberrant anticlinal cell divisions in the pericycle in a RGF RECEPTOR (RGFR)-dependent manner (Fernandez et al. 2013a, 2015, 2020; Jourquin et al. 2022, 2023). Further studies suggest that RGF peptide signaling maintains the proper RAM stem cell niche through modulating the expression and stability of PLETHORA1 (PLT1) and PLETHORA2 (PLT2) proteins (Matsuzaki et al. 2010; Shinohara et al. 2016; Ou et al. 2016). Additionally, a transcription factor, RGF1-INDUCIBLE TRANSCRIPTION FACTOR 1 (RITF1), is shown to be induced by RGF1 to increase superoxide anion levels, eventually enhancing the PLT2 protein stability (Yamada et al. 2020). Recently, it is found that low extracellular pH promots RGF signaling to facilitate plant growth (Liu et al. 2022b). Specifically, extracellular acidity stimulats the interaction between RGF1 and the receptor RGI1, leading to immunity suppressiong and growth promotion (Liu et al. 2022b).

RGF peptides are also involved in many other plant growth and developmental processes (Fernandez et al. 2013b; Jourquin et al. 2020). RGF1 application inhibits lateral root formation. Similarly, the *rgf5 rgf8* double mutant exhibits increased densities of lateral root primordium (Fernandez et al. 2020; Jourquin et al. 2022, 2023). Furthermore, MITOGEN-ACTIVATED PROTEIN KINASE 6 (MPK6) has been implicated in RGF-regulated lateral root development (Fernandez et al. 2013a, 2015, 2020). Moreover, the *rgf* mutant and transgenic lines overexpressing *RGF* genes display defective root hair development (Fernandez et al. 2013a). Additionally, overexpression of *RGF6*, *RGF4*, and *RGF9*, or treatment with these peptides, disrupts gravitropic bending in both roots and hypocotyls (Whitford et al. 2012; Xu et al. 2023a). Knock-down the *RGF3* (*MtRGF3*) gene in *M. truncatula* resulted in an increased number of root nodules. Conversely, *RGF3*-overexpression plants and those treated with synthesized MtRGF3 peptides exhibited decreased nodule numbers and inhibited lateral root development (Li et al. 2020). RGF peptides also participates in peach ripening (Tadiello et al. 2016; Busatto et al. 2017).

Induced by *Pseudomonas syringae*, RGF7 confers resistance to the pathogen (Wang et al. 2021b; Stegmann et al. 2022). *RGF7* overexpressors enhance defense response and disease resistance to pathogens, thus acting as an endogenous amplifier in plant immunity dependent on leaf-expressed RGI4 and RGI5 receptors (Wang et al. 2021b). The expression of *RGF6* and *RGF9* is down-regulated upon infected by *P.syringae*. Furthermore, peptide application, overexpression and loss-of-function studies demonstrated that RGF6 and RGF9 are positive regulators against bacterial pathogens (Stegmann et al. 2022). Mechanistically, RGF6 is perceived by RGI3 which could form a receptor complex with FLS2 (Stegmann et al. 2022). In addition, RGF6-RGI3 signaling promotes posttranscriptional FLS2 protein abundance (Stegmann et al. 2022). Thus, the RGF-RGI signaling controls pattern-triggered immunity.

### Inflorescence Deficient in Abscission (IDA) peptides

The primary role of IDA and IDA-LIKE (IDL) peptides is to trigger abscissions of different organs across diverse plants (Wang et al. 2023a) (Fig. 2). In principle, IDA peptides are recognized by HAESA (HAE) and HAESA-LIKE 2 (HSL2) receptor kinases, thereby stimulating downstream signaling that leads to cell separations (Santiago et al. 2016). The *ida* mutant shows delayed floral abscission phenotype, whereas *IDA*/*IDL* overexpression transgenic plants exhibit ectopic abscissionin various organs (Butenko et al. 2003; Stenvik et al. 2008; Wang et al. 2023a). Furthermore, IDA/IDLs also participate in stress-induced abscission: drought and *Pseudomonas syringae* DC3000 trigger leaf abscission that is defective in *ida* mutants (Patharkar and Walker 2015, 2016). The abscission-regulated role of IDA/IDL peptides has been observed in multiple plant species, including *Nicotiana benthamiana* (Ventimilla et al. 2020, 2021; Guo et al. 2021), *Lupinus luteus* L. (Wilmowicz et al. 2021), and rose (Singh et al. 2023). Applications of synthetic IDA/IDL peptides have also been shown to trigger ripen fruit abscission in oil palm (Tranbarger et al. 2019) and mango (Rai et al. 2021). Treatments of *Populus* with synthetic IDA or IDL1 peptides enhanced dark-induced leaf abscission (Stø et al. 2015; Tranbarger et al. 2019). Moreover, a mutation in the tomato *SlIDL6* gene leads to delayed flower abscission under low light stress (Li et al. 2021).

IDA/IDLs also regulate different aspects of plant growth and developmental plasticity (Wang et al. 2023a). The *ida* mutant shows significantly decreased lateral root density (Kumpf et al. 2013). Plants with enhanced *IDL1* signals through HSL2 show increased frequency of sloughing and initiation of new cell layers in the root cap (Shi et al. 2018). Furthermore, *IDL6* has been shown to regulate dark-induced senescence (Guo et al. 2022). In soybean, overexpression of *GmIDL2a* or *GmIDL4a* increased lateral root density (Liu et al. 2018). Knockout of *SlIDA* in tomato resulted in a severe defect in male gametes, leading to decreased pollen germination and pollen tube elongation (Wang et al. 2020b).

IDA/IDLs have also been shown to participate in plants’ responses to biotic and abiotic stresses. A variety of biotic and abiotic signals can induce *IDA* expression (Lalun et al. 2024), while pathogens and UV light trigger the transcription of *IDL6* and *IDL7* (Vie et al. 2015, 2017). Interestingly, IDA can induce signatures of early defense responses, such as ROS and cytosolic calcium ions, thus protecting tissues undergoing cell separation from pathogen attacks (Butenko et al. 2014; Lalun et al. 2024). Plants overexpressing *IDL6* show more severe disease symptoms than WT plants after *P. syringae* DC3000 infection; while knockdown of *IDL6* increased resistance to this pathogen (Wang et al. 2017c). Analyses of *cis*-acting regulatory elements reveal that several *Nicotiana benthamiana IDL* genes contain drought response elements in their promoter regions, and their expressions can be induced by drought stress (Ventimilla et al. 2020). In tobacco, numerous stress-related *cis*-elements have also been identified in the promoters of *NtIDL*s, and their expressions are induced by salt and wounding stresses (Guo et al. 2021).

### Casparian Strip Integrity Factor (CIF) peptides

CIF peptides play essential roles in the formation of the Casparian strip, embryonic cuticle, and pollen wall. The *Arabidopsis thaliana* and rice *cif* mutants display defects in endodermal barriers and continuity of the Casparian strip in roots (Nakayama et al. 2017; Zhang et al. 2024a) (Fig. 2). Following transmission through the Casparian strip, CIF1/CIF2 peptides are sensed by GASSHO1 (GSO1) and GSO2 receptors to trigger the completion of the Casparian strip formation. In return, the completed Casparian strip restricts the transferring of CIF1/CIF2 outward into endodermal tissue and thus terminate the signal (Nakayama et al. 2017; Doblas et al. 2017; Fujita et al. 2020). The *cif1 cif2* double mutant is shown to be hypersensitive to excess iron concentrations (Nakayama et al. 2017). Conversely, mutation of *CIF2* increases sensitivity to low K^+^ conditions (Wang et al. 2021a). Loss of *CIF3* and *CIF4* simultaneously leads to the formation of large, misshapen, fused pollen grains (Truskina et al. 2022). TWISTED SEED1 (TWS1) is also a CIF-like peptide, characterized by the presence of a CIF domain in its protein. The *tws1* mutant exhibits a variety of growth defects at different developmental stages, in particular, with cup-shaped cotyledons, short hypocotyl, short siliques, twisted seed shape, and defective cuticle of embryos (Fiume et al. 2016; Doll et al. 2020).

### Epidermal Patterning Factor (EPF)/Stomagen peptides

The EPF/EPF-LIKE (EPFL)/STOMAGEN family peptides act as cell-to-cell signals for diverse biological processes (Zeng et al. 2020) (Fig. 2). Mutations of *EPF1/EPF2* genes lead to increased stomatal density, while overexpression of *EPF1/EPF2* significantly reduced stomatal density (Hara et al. 2007, 2009). In contrast, EPFL9/STOMAGEN is shown to promote stomatal differentiation (Hunt et al. 2010). EPF1 and EPF2, and EPFL9 have been shown to act as ligands for ERECTA and ERECTA-LIKE 1 (ERL1) receptors in stomatal development, although exerting different effects. As such, EPFL9/STOMAGEN competitively replaces EPF2 binding to ER, leading to fine-tune stomatal patterning (Lee et al. 2015). EPFL4 and EPFL6 peptides regulate procambial cell division and stem elongation (Ikematsu et al. 2017; Fischer and Teichmann 2017). EPFL4, EPFL5, and EPFL6 peptides also promote stamen filament elongation to ensure self-pollination under both normal and cold temperatures conditions (Negoro et al. 2023; He et al. 2023). EPFL2 and EPFL9 collectively coordinate in ovule initiation which controls seed number and fruit size (Kawamoto et al. 2020). EPFL1-EPFL6 are also redundantly involved in integument elongation in ovules (Li et al. 2023). Furthermore, EPFL peptides control female germline specification (Cai et al. 2023). Overexpression of *EPF* homologs in rice reduces plant height, and increases SAM width, indicating their roles in organ elongation (Mohammed et al. 2019). In rice, down-regulation of *GRAIN LENGTH AND AWN DEVELOPMENT1* (*GAD1*)/*OsEPFL1/OsEPFL2* transcript levels leads to increased grain number, shorter grains lengths, smaller grain sizes, and shorter awns (or awnless) phenotypes (Jin et al. 2016; Xiong et al. 2022), as well as decreased seed germination rates (Jin et al. 2023). Notably, mutations in rice *EPF*/*EPFL* members, including *OsEPFL6*, *OsEPFL7*, *OsEPFL8*, and *OsEPFL9*, heavily impaires rice panicle morphology, spikelet number, spikelet fertility, and grain yield (Guo et al. 2023).

### Phytosulfokine (PSK) peptides

PSKs are sulfated pentapeptides that regulate cell divisions through perceiving by PSK RECEPTOR-1 (PSKR1) and PSKR2 receptors (Matsubayashi and Sakagami 1996; Matsubayashi et al. 2002). Consistently, PSKs play roles in various biological processes such as plant growth, development, and defense response (Shen et al. 2023a) (Fig. 2). Studies have demonstrated that PSKs enhance callus cell proliferation (Eun et al. 2003), promote adventitious root growth (Yamakawa et al. 1998), facilitate somatic embryogenesis (Hao et al. 2023), and root elongation (Reichardt et al. 2020), stimulate pollen tube growth (Stührwohldt et al. 2015; Kou et al. 2020), and maintain procambial cell identity (Holzwart et al. 2018). For instance, many studies highlight the effects of PSK peptides in plant tissue culture of diverse plant species (Yamakawa et al. 1998; Yang et al. 1999; Hanai et al. 2000; Kutschmar et al. 2009; Asif et al. 2014; Ochatt et al. 2018; Wu et al. 2019; Joo et al. 2022). In the root quiescent center, PSK’s role in regulating root elongation is controlled by ETHYLENE RESPONSE FACTOR 115 (ERF115), a crucial regulator for cell division (Heyman et al. 2013; Kong et al. 2018). To fulfill their functions, PSK peptides often induce a rapid increase in the level of CYCLIC GUANOSINE MONOPHOSPHATE (cGMP), which is involved in regulating growth and defense responses (Kwezi et al. 2011). Additionally, PSKs have been shown to be involved in seed size and yield in soybean crops (Yu et al. 2019a).

In contrast to stimulating cell division, PSKs appear to attenuate plant stress responses. The application of exogenous PSK peptides or overexpression of the *PSK* precursor genes impairs resistance to (hemi)biotrophic bacterial pathogens (Mosher et al. 2013; Rodiuc et al. 2016). Furthermore, tomato PSKs are shown to be implicated in organ abscission upon drought stress conditions (Reichardt et al. 2020). Furthermore, PSKs effectively balance growth and defense responses in tomato (Ding et al. 2023). The overexpression of *PSK1* has been shown to result in remarkedly enhanced osmotic stress resilience (Stührwohldt et al. 2021). These data suggest the potential of PSK peptides as candidates for agricultural applications. Indeed, further studies have shown that the application of PSKs in broccoli and strawberries delays the senescence and lengthened the storage time (Aghdam et al. 2020; Aghdam and Alikhani-Koupaei 2021; Aghdam and Flores 2021).

### Plant Peptides Containing Sulfated Tyrosine (PSY) peptides

PSY peptides undergo PTMs including tyrosine sulfation and hydroxyproline arabinosylation (Fig. 2). In the *Arabidopsis thaliana* genome, 9 members of the *PSY* genes have been identified (Ogawa-Ohnishi et al. 2022). PSY peptides impact various aspects of plant biological processes, particularly cell proliferation, root growth, and the trade-off between plant growth and stress adaption (Amano et al. 2007; Mosher et al. 2013; Shen and Diener 2013; Ogawa-Ohnishi et al. 2022). The PSY1 peptide, functioning antagonistically to RALF, promotes the acidification of the apoplastic space (Fuglsang et al. 2014; Gjetting et al. 2020). While the *psy5 psy6 psy8* triple mutant does not display an apparent phenotype, *35 S::PSY1* transgenic seedlings develop longer roots and larger cotyledons. Similarly, the application of synthetic PSY peptides exerts growth promotion (Amano et al. 2007; Ogawa-Ohnishi et al. 2022). Treatments of wild-type plants with PSY5 peptide or overexpression of *PSY6* lead to decreased salt tolerance (Ogawa-Ohnishi et al. 2022). Notably, *Xanthomonas* RaxX peptides are highly similar to PSY family peptides, sharing a similar function in promoting root development (Pruitt et al. 2015, 2017). However, RaxX, unlike PSYs, triggers the immune response in rice via directly perceiving by the immune receptor XA21 (Pruitt et al. 2015; Luu et al. 2019). In addition to the rice pathogen *Xanthomonas*, root-knot nematodes (*Meloidogyne* spp.) haven also been shown to secrete PSY-like peptides to manipulate the immune system of plants (Yimer et al. 2023).

### Pamp-Induced Secreted Peptide (PIP) peptides

PIP peptides were initially discovered due to their significant inductions by a variety of pathogens and elicitors including exogenous PAMPs like flagellin and chitin (Hou et al. 2014) (Fig. 2). Eleven PIP/PIP-LIKEs (PIPLs) peptide-coding genes have been identified in the *Arabidopsis thaliana* genome, and these peptides have been shown to play crucial roles in plant growth and development, as well as in abiotic and biotic stress tolerance (Hou et al. 2014; Vie et al. 2015). Exogenous application of PIP1 and PIP2 peptides, or overexpression of their precursor genes, significantly activated PAMP-triggered immunity (PTI) responses, encompassing ROS bursts and the modulation of defense-related gene expression, thereby leading to augmented immune responses and increased pathogen resistance (Hou et al. 2014). Seedlings treated with synthetic PIP peptides also display a reduction of primary root elongation (Hou et al. 2014; Yu et al. 2023b). Transgenic plants overexpressing *PIP2* and *PIP3* display shorter primary roots, while the *pip2* mutant displays no obvious root defects. The *pip3* mutant and the *pip2 pip3* double mutant both exhibit a short primary root phenotype (Hussain et al. 2021). *PIPL3* overexpressing plants exhibit a decreased number of lateral roots, but the length of primary roots remains unaffected (Toyokura et al. 2019). Conversely, overexpression of *PIP2* stimulats hypocotyl elongation, but the hypocotyl lengths of *35 S::PIP3*, *pip2*, *pip3*, and *pip2 pip3* mutants remain unchanged (Hussain et al. 2021). When infected by *P. syringae* and *Botrytis cinerea*, *PIP3* loss-of-function mutant plants exhibit no obvious phenotype. However, plants overexpressing *PIP3* show increased sensitivities to both pathogens (Najafi et al. 2020). Additionally, PIP3 has been shown to regulate plant immunity by modulating crosstalk between salicylate and jasmonate signaling pathways (Najafi et al. 2020). Homologous PIP1 proteins are identified in potatoes (*Solanum tuberosum*), in which they enhance plant resistance against potato virus Y (PVY) infection (Combest et al. 2021).

### Plant Elicitor Peptide (PEP) peptides

PEP peptides contribute to defense responses against pathogen attack and abiotic stress in plants (Zelman and Berkowitz 2023) (Fig. 2). Generally, *PEP*s are induced by wounding, jasmonic acid, and pathogens (Yamaguchi et al. 2006, 2010). Treatments with PEP peptides or overexpression of the *Arabidopsis thaliana PEP* precursor gene enhance disease resistance (Huffaker et al. 2006; Yamaguchi et al. 2006, 2010). Under the alkaline environment, immunity responses stimulated by the PEP1-PEPR signaling module are enhanced, while the RGF1signaling pathway is inhibited to fine-tune growth and immunity (Liu et al. 2022b). Importantly, PEP peptides significantly induce stomatal closure, highlighting the significance of the PEP peptide signaling in stomatal immunity (Qu et al. 2019). Further investigations have revealed that PEP-induced stomatal immunity acts through SLAC1 and SLAH3 activation in an OST1-independent manner (Zheng et al. 2018). While PEP1 peptide treatments represses primary root growth (Jing et al. 2019), overexpression of the *PEP3* gene or application of the PEP3 peptides increases plants tolerance to salinity (Nakaminami et al. 2018). PEP members identified in other species such as maize, soybean, and rice also play a conserved role in defense against various pathogens (Zelman and Berkowitz 2023).

In addition, PEP peptides have been shown to play important roles in plant growth and development. The *Arabidopsis thaliana pep7* mutant displays a reduced number of lateral root primordia at various developmental stages, while treatments with the PEP7 peptide promotes lateral root primordia formation during these stages (Wang et al. 2022b). More recently, the tomato PEP1 peptide, REGENERATION FACTOR1 (REF1), is shown to function as a regulator of wound-induced cellular reprogramming and organ regeneration in plants (Yang et al. 2024).

### Serine Rich Endogenous Peptide (SCOOP) peptides

SCOOPs are serine-rich peptide hormones, and 50 putative members have been identified in *Arabidopsis thaliana* (Yang et al. 2023) (Fig. 2). SCOOP12, the founding member of this family, plays a role in immune response and root growth regulation. The *scoop12* mutant exhibits significantly longer primary roots, while applications of the SCOOP12 peptide induces a dose-dependent decrease in primary root growth (Gully et al. 2019; Guillou et al. 2022b).Treatments of plants with the SCOOP12 peptide trigger a wide range of short- and long-term immune responses, and the *scoop12* mutant shows stronger resistance to pathogens (Gully et al. 2019). Similarly, applications of several other SCOOP peptides also stimulate immune responses (Hou et al. 2021). On the other hand, the SCOOP10 peptide did not show any effects on root growth inhibition. However, mutation of the *SCOOP10* gene leads to earlier flowering (Guillou et al. 2022b). A recent study also suggested that SCOOP10 and SCOOP12 act as antagonistic peptides in regulating leaf senescence through the receptor MALE DISCOVERER 1-INTERACTING RECEPTOR-LIKE KINASE 2 (MIK2) (Zhang et al. 2024c).

### Non-canonical peptides

Over the past decades, most characterized plant peptides are derived from larger precursor proteins. However, with next-generation sequencing, accumulating evidence has revealed a notable abundance of non-canonical peptides, originating from previously recognized non-coding regions, including intergenic regions, 5’ untranslated regions (UTRs), 3’ UTRs, intronic regions, and various genomic junctions (Couso and Patraquim 2017; Wang et al. 2020c; Sami et al. 2024). Numerous examples of non-canonical peptides highlight their importance in diverse biological processes. For instance, the first lncRNA-encoded peptide, EARLY NODULIN 40 (ENOD40), initially identified in soybean (Yang et al. 1993), promotes soybean nodulation through nitrogen availability (Xu et al. 2021; Yun et al. 2022). In maize, some non-canonical peptides from introns and intergenic regions are characterized for their role against fungal pathogens (Tian et al. 2021). The intergenic product POLARIS regulates root growth and leaf vascularization (Casson et al. 2002), while another intergenic peptide, ROTUNDIFOLIA4, contributes to leaf morphogenesis in *Arabidopsis thaliana* (Narita et al. 2004). Similarly, 575 oxidative stress-induced peptides (OSIPs), primarily from intergenic regions but also from pseudogenes and gene introns, have been identified in *Arabidopsis thaliana.* In *Physcomitrella patens*, mass spectrometry confirmed the translation of 9 sORFs located on lncRNA. *Physcomitrella patens* sORF encoded peptide 1 (PSEP1) (41-aa) is linked to aging and cell death, while, PSEP3 (57-aa) is involved in filament branching. Additionally, PSEP25 influences protonemal architecture and leafy shoot count, while PSEP18 (40-aa) is associated with impaired growth rate (Fesenko et al. 2019).

In addition to the non-canonical peptide types mentioned above, recent research suggests that primary microRNAs (pri-miRNAs) may contain sORFs that encode micropeptides, known as miPEPs. miPEP165a, the first pri-miRNA encoded peptide discovered in plants, promotes meristem cell proliferation and increases primary roots length in *Arabidopsis thaliana* through exogenous application or overexpression (Lauressergues et al. 2015). Several other pri-miRNA-encoded peptides, including miPEP171d, miPEP172c and miPEP858a, are also implicated in plant growth and development. For instance, miPEP171d1 in grapes promotes adventitious roots formation (Chen et al. 2020) (Fig. 2), while miPEP172c enhances nodulation in soybean without altering other root structures (Couzigou et al. 2016). In *Arabidopsis thaliana*, exogenous application of miPEP858a rescues developmental defects in mutants, including impaired growth and delayed flowering (Sharma et al. 2020). In an in vitro assay, 23 of 87 synthetic miPEPs tested were found to affect root development in *Arabidopsis thaliana* seedlings (Ormancey et al. 2021). Among these, AtmiPEP164b, a strong root growth inhibitor, and AtmiPEP397a, a strong promoter of root growth, were identified. Their homologs, BvmiPEP164b and BomiPEP397a, displayed similar phenotypes in *Brassica oleracea* and *Barbarea vulgaris*, demonstrating the functional conservation of miPEPs across species (Ormancey et al. 2021). Recently, a 31-amino-acid micropeptide derived from a sORF specific to the Zea genus, named microRPG1 (qKDR1 Regulated Peptide Gene), was discovered to negatively regulate kernel dehydration rate by modulating the activity of ethylene signaling components, ZmETHYLENE-INSENSITIVE3-like 1 and 3 in maize (Yu et al. 2024).

## Receptor-like kinase-mediated peptide sensing

Cell-to-cell and cell-to-environment communications play critical roles for plants to respond to environmental changes. During this process, plants generate diverse signaling molecules that trigger intricate signaling cascades, enabling them to effectively acclimate to environmental fluctuations. These signaling molecules include plant hormones and small peptides (Bari and Jones 2009; Chevalier et al. 2011; Yue and Beeckman 2014; Ku et al. 2018; Takahashi and Shinozaki 2019; Chen et al. 2021; Tabassum and Blilou 2022; Xie et al. 2022a). As a new type of plant hormones, small peptides serve as vital signals to communicate between cell and cell, or between cell and environment, to regulate many aspects of plant growth, development, reproduction, and defense responses (Katsir et al. 2011; Hirakawa and Sawa 2019; Ogawa-Ohnishi et al. 2022; Lan et al. 2023).

Peptide hormones are usually perceived by receptor-like protein kinases (RLKs), a superfamily of transmembrane proteins that typically have an extracellular domain, a single transmembrane domain, and a cytoplasmic kinase domain, and relay the signals into the cell to regulate gene transcriptions and protein translations (Zhu et al. 2024). The RLK-mediated signaling may also directly regulate activities of transporters and channels on the plasma membrane to regulate cellular responses (Hajný et al. 2020; Lin et al. 2021; Li et al. 2022c). Hundreds of RLKs have been identified in plants, which mediate various signals. For example, more than 610 RLKs have been identified in *Arabidopsis thaliana* (Shiu and Bleecker 2001a, b; Zhu et al. 2024), and over 1000 in rice (Zhu et al. 2024). The extracellular peptide signals are either perceived by preformed complexes of RLKs, or multiple RLKs undergoing dimerization or polymerization after sensing the peptides, either with themselves, or with co-receptor RLKs carrying a shorter extracellular domain, or other membrane-anchored proteins, leading to autophosphorylation and enhanced transphosphorylation (Li et al. 2015; Song et al. 2017; Hu et al. 2018; Gou and Li 2020). Phosphorylated RLKs then proceed to phosphorylate downstream signaling molecules such as MAPKs. Through a cascade of signaling events, MAPKs amplify the signal and subsequently phosphorylate downstream kinases or transcription factors, ultimately triggering cellular responses to regulate plant growth and development, reproductive development, immune responses, and many other processes (Cristina et al. 2010; Li et al. 2019b; Wang and Gou 2020).

### CLV1 and BAMs mediate CLE signals to regulate stem cell fate

Peptide hormones are usually perceived by leucine-rich repeat family RLKs (LRR-RLKs). Efforts have been made to decipher the role of CLE peptides in a variety of biological processes in *Arabidopsis thaliana* and other plant species (Cock and McCormick 2001; Fletcher 2020; Song et al. 2022b). Loss-of-function of *CLV3* led to increased stem cell numbers, enlarged SAM, and more floral organs (Clark et al. 1995). *CLV3* encodes a small extracellular protein and regulates stem cell homeostasis in the SAM (Fletcher et al. 1999) through a small peptide corresponding to the CLE motif near C-terminal region (Fiers et al. 2005). The CLV3 signal is perceived by CLV1, a LRR-RLK, to maintain SAM homeostasis (Clark et al. 1997). Several studies showed that BAMs, homologous members of CLV1, also play important roles in regulating meristem homeostasis (DeYoung et al. 2006; Gao and Guo 2012). The *bam1/2/3* triple mutants exhibited fewer floral organs and smaller inflorescence meristems (DeYoung et al. 2006; DeYoung and Clark 2008). BAM1 also perceives CLE40 signal, and functions together with the CLV3–CLV1 signaling pathway to coordinate stem cell activity in the central zone with cell differentiation activity in the periphery zone of the SAM (Schlegel et al. 2021). In addition, BAMs mediate CLE signals to regulate stem cell division and differentiation in roots. CLE13/16 act as ligands of BAMs to control formative division of the cortex/endodermis initial (CEI). Loss-of-function of *BAM1/2* resulted in one ground tissue layer in the mutant root (Crook et al. 2020). CLE9/10 are sensed by BAM1/3 to regulate periclinal division of the xylem initial cells, as *cle9* and *bam1/3* null mutants produced more xylem cells (Kondo et al. 2011; Qian et al. 2018). CLE25/26/45 peptides act together to inhibit BAM1/3-mediated phloem cell differentiation. Consistently, *cle25/26/45* and *bam1/2/3* mutants exhibited increased phloem cells (Depuydt et al. 2013; Hazak et al. 2017; Hu et al. 2022; Qian et al. 2022). In roots, CLE40 is also sensed by ACR4, a non-LRR-RLK, to regulate the fate of columella stem cells (Stahl et al. 2009).

### CrRLK1Ls mediate RALF signals to regulate plant growth and reproduction

RALF family peptides have been found to induce extracellular alkalinization and inhibit cell growth. RALFs are primarily sensed by a CrRLK family receptor kinase which is named after the first member characterized in *Catharanthus roseus* cell cultures (Schulze-Muth et al. 1996). In *Arabidopsis thaliana*, the CrRLK1-like (CrRLK1L) family receptor kinases consist of 17 members, and are featured with a conserved structure with one or two extracellular malectin-like domains (MLD), a transmembrane domain and an intracellular kinase domain (Li et al. 2015). Among these CrRLK1Ls, the FER receptor kinase was first discovered to bind to the RALF1 peptide, which is highly expressed in roots (Haruta et al. 2014). FER was initially characterized as a regulator of female fertility, as mutation of *FER* led to pollen tube overgrowth within the embryo sac, failure in rupture and sperm cell release, resulting in female semi-sterility and polyspermy (Huck et al. 2003; Hématy et al. 2007; Duan et al. 2020). The RALF1-FER interaction results in the phosphorylation of the plasma membrane H^+^-ATPase2 at Ser899, mediating the inhibition of proton transport and cell expansion (Haruta et al. 2014). Beyond FER, other CrRLK1L family members also regulate downstream signaling pathways, modulating plant growth and development through sensing different RALFs. The ANJ–FER receptor kinase complex recognizes autocrine RALF23 and RALF33 in stigmatic papillary cells, triggering the production of ROS via the ROP2‒RBOHD pathway. This process acts as a defensive mechanism to block undesirable pollen and pathogen spores (Liu et al. 2021a). Moreover, RALF4 and RALF19 peptides engage with the CrRLK1Ls of ANXUR1/2 (ANX1/2) and Buddha’s Paper Seal1/2 (BUPS1/2) to maintain pollen tube integrity. Conversely, a shift in binding to RALF34 resulted in pollen tube rupture and sperm release (Ge et al. 2017; Mecchia et al. 2017).

### Receptor and co-receptor complexes underlying peptide hormone perception

Collaboration between receptors and co-receptors of LRR-RLKs is usually required to sense small peptide signals and transmit them into the cell to regulate different biological processes (Ma et al. 2016; Gou and Li 2020). For example, RGI/RGFR and SOMATIC EMBRYOGENESIS RECEPTOR-LIKE KINASE (SERK) form a receptor–co-receptor complex to sense extracellular RGF peptide signal to regulate stem cell homeostasis in the RAM (Shinohara et al. 2016; Song et al. 2016; Ou et al. 2016, 2022; Lu et al. 2020). The sulfated TWISTED SEED1 (TWS1) peptide is perceived by receptor GSO1/2 and co-receptor SERKs to regulate the integrity of embryonic cuticle (Tsuwamoto et al. 2008; Fiume et al. 2016; Doll et al. 2020; Zhang et al. 2022a). Bacterial FLAGELLIN 22 (flg22) binds to the FLS2-SERK receptor complex to regulate plant immune response (Gómez-Gómez and Boller 2000; Asai et al. 2002; Chinchilla et al. 2007; Sun et al. 2013). CIKs, a group of co-receptors discovered in recent years, play a crucial role in determining anther cell fate. Loss-of-function of *CIKs* results in aberrant anther locule formation and more microspore mother cell-like cells, resembling phenotypes observed in *bam1/2* anthers. Genetic analysis revealed that CIKs function with BAM1/2 in a common genetic pathway to control anther cell differentiation. CIKs interact with, and are phosphorylated by BAM1/2, supporting their roles as co-receptors of BAM1/2 during early anther development (Hord et al. 2006; Cui et al. 2018). A striking defect observed in *cik* mutants is their extremely enlarged and fasciated SAM, a typical phenotype observed in *clv* mutants (Clark et al. 1997; Kayes and Clark 1998; Brand et al. 2000; Kinoshita et al. 2010; Hu et al. 2018). Genetic and biochemical results indicated that CIKs act as co-receptors of CLV1, CLV2, RECEPTOR-LIKE PROTEIN KINASE 2 (RPK2) to sense the CLV3 signal for maintaining SAM homeostasis (Hu et al. 2018). Additionally, CIKs are also recruited as co-receptors of ACR4 to sense the CLE40 signal in maintaining stem cell homeostasis of the distal RAM (Zhu et al. 2021b).

### BAM1/3–CIK receptor complex percieives CLE25/26/45 signals to inhibit protophloem differentiation in roots

**Fig. 3:**
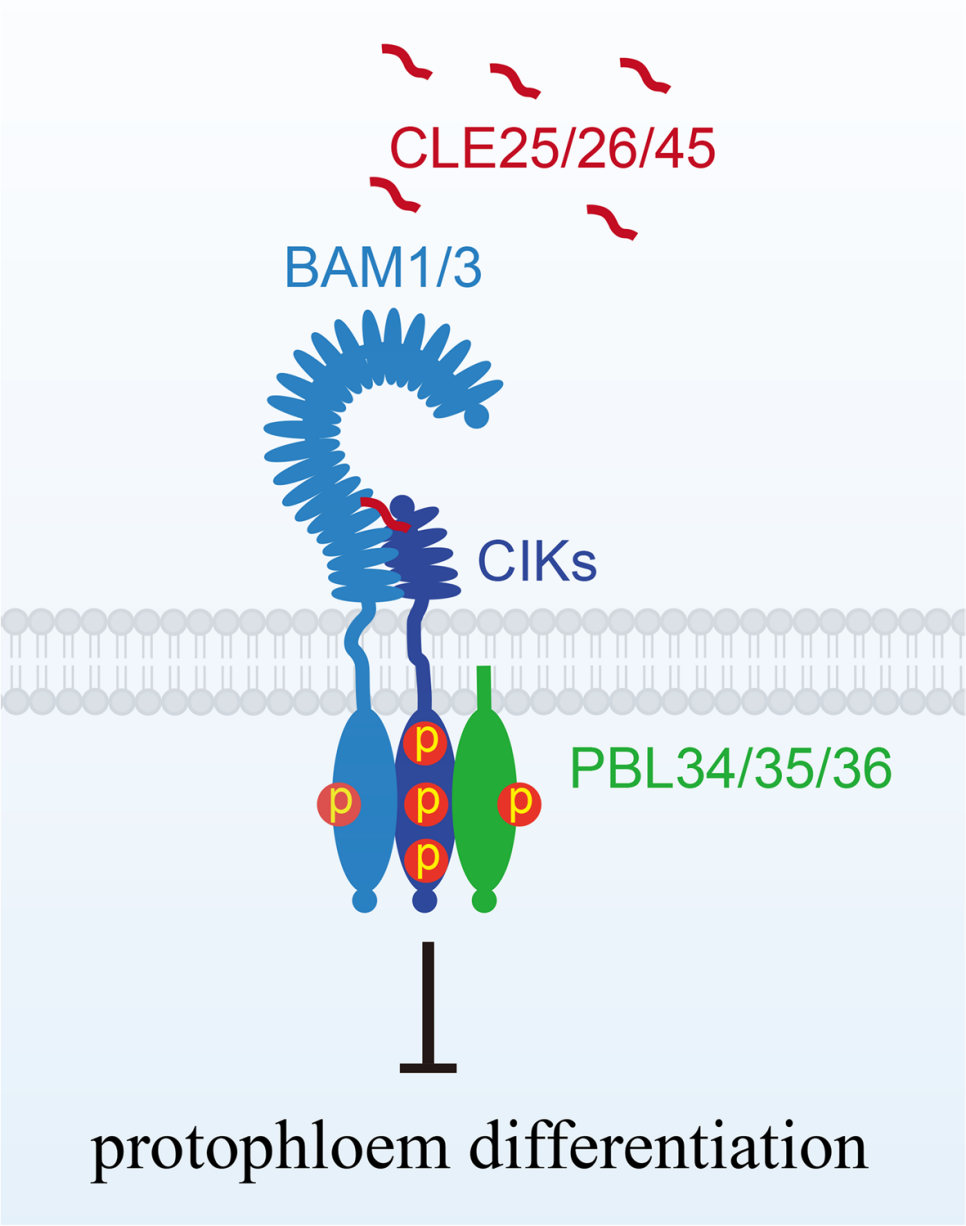
The CLE–BAM–CIK signaling pathway regulates protophloem differentiation. Perception of CLE 25/26/45 ligands by the extracellular domain of BAM1/3 receptors located on the plasma membrane triggers the interaction between BAM1/3 and CIKs. The BAM–CIK receptor complex then undergoes phosphorylation. The phosphorylated receptor complex subsequently leads to phosphorylation of PBL34/35/36, mediating downstream signaling events to regulate protophloem differentiation and maintain root meristem homeostasis

In recent years, mechanisms of CLE signaling pathway in regulating phloem cell differentiation and RAM homeostasis have been revealed in detail. CLE25, CLE26, CLE33 and CLE45 are involved in the formation of phloem in plants. The *brevis radix* (*brx*) and *octopus* (*ops*) mutants exhibited a short-root phenotype because protophloem cells were not correctly differentiated during root development (Mouchel et al. 2004; Truernit et al. 2012). A suppressor screening identified a *bam3* mutation that can rescue the defects of protophloem development in *brx* mutant (Depuydt et al. 2013). In addition, *bam3* mutants exhibited reduced sensitivity to CLE45 peptide application, while in vitro treatment with CLE45 peptide inhibited both phloem differentiation and root growth in the wild type. These results suggest that CLE45 peptide acts as a ligand of BAM3 to coordinately inhibit protophloem differentiation (Depuydt et al. 2013). Because *bam3* mutant showed partial insensitivity to CLE45 peptide treatment, the *bam1/3* double mutants were investigated and they were completely insensitive to CLE45 peptide, suggesting that BAM1/3 redundantly suppress protophloem differentiation in roots (Hu et al. 2022). It was reported that homologous BAM1 and BAM3 can ectopically compensate for the loss-of-function of CLV1 to regulate SAM homeostasis (Nimchuk et al. 2015). Moreover, CIK1/2/3/4 are recruited as co-receptors of CLV1 to regulate SAM homeostasis. These results together suggested that CIK members may also play critical roles in regulating protophloem differentiation through CLE45–BAM1/3 signaling. As expected, the high-order *cik2/3/4/5/6* mutants exhibited insensitivity to CLE45 peptide treatment, and *cik2/3/4/5/6* mutations suppressed protophloem development defects and the short-root phenotype of *brx* and *ops*, similar to *bam1/3* mutations (Hu et al. 2022). Combined expressional, phylogenetic, and physiological analyses identified CLE25/26 that function together with CLE45 to regulate phloem differentiation, as *cle25/26/45* triple mutants showed earlier differentiation of protophloem cells. Similarly, *cle25/26/45* mutations suppressed the defects of protophloem cells in *brx* and *ops* root tips. Applications of these three CLE peptides enhanced phosphorylation of CIKs and promoted interactions between CIKs and BAM3. Importantly, elevated phosphorylation levels of CIKs upon CLE25/26/45 peptide applications are dependent on BAM1/3. These findings demonstrate that CIKs serve as co-receptors of BAM1/3 to perceive the CLE25/26/45 signals during protophloem differentiation in roots (Hu et al. 2022) (Fig. 3).

### LRE/LLGs act as chaperones and co-receptors of CrRLK1Ls

In the studies of CrRLKL receptor kinases, a novel co-receptor has been identified, namely the glycosylphosphatidylinositol (GPI)-anchored protein LORELEI (LRE) that acts as a functional co-receptor. The signaling pathways associated with CrRLK1Ls encompass notable interactions with the LRE GPI-anchored protein and its relatives, LLG1-3 as chaperones and co-receptors for FER and its homologs ANX1/2 and BUPS1/2 (Capron et al. 2008; Li et al. 2015; Feng et al. 2019). GPI-anchored proteins (GAPs) distinguish themselves by being tethered to the outer leaflet of the lipid bilayer through a GPI moiety, rather than spanning the membrane (Fivaz et al. 2002). The synthesis of the GPI anchor occurs in a complex multistep process within the ER membrane, with these GAPs subsequently sorted to the plasma membrane surface via the trans-Golgi network (TGN) by clustering within sphingolipid- and cholesterol-rich microdomains, or rafts (Orlean and Menon 2007; Zurzolo and Simons 2016). Li et al. illustrated the novel role of LLG1/LRE in the FER receptor’s sensing of RALF1(Li et al. 2015). They detailed how LLG1/LRE attaches to FER’s extracellular juxtamembrane region both in the ER and on the cell surface. Absent LLG1, FER’s journey to the plasma membrane is hindered, trapping significant amounts of it within the ER. Moreover, LLG1’s interaction with the FER on the plasma membrane to perceive RALF1 peptide, which induces the downstream ROPGEF–RAC/ROP-ROS signaling, making LLG1 a crucial chaperone for FER’s membrane transport and a coreceptor for the RHO GTPase signaling regulated by FER. Structural biology studies further substantiated the direct binding of LLG2 to the conserved N-terminal region of RALF23, emphasizing the role of LLGs’ conformationally flexible C-terminal sides in the selective recognition of RALF23. This specificity underscores the significance of LLGs in the FER-dependent perception of RALF23 in *Arabidopsis thaliana* (Xiao et al. 2019).

Further studies highlighted the pivotal role of LRE and LLGs in enhancing the cell surface signaling of CrRLK1Ls for RALFs (Ge et al. 2019; Galindo-Trigo et al. 2020). The complex formed between RALF1, LLG1, and FER, through RopGEF1 activation, initiates RAC/ROPs, which in turn recruit NADPH oxidase. This cascade is vital for regulating ROS production, crucial for root hair growth (Duan et al. 2010; Li et al. 2015). Subsequent investigations revealed LLG2/3’s roles as chaperones and coreceptors alongside ANX1/2 and BUPS1/2 for pollen tube growth. CRISPR-generated *llg2/3* mutants, exhibiting severe fertility defects analogous to *anx1/2* and *bups1/2* mutants, further demonstrated this point (Ge et al. 2019). These *llg2/3* knockdown mutants presented with shorter and more frequently bursting pollen tubes (Feng et al. 2019). The interaction between LLG2/3 and ANX/BUPS, modulated by RALF4 concentration, is key to secreting ANX/BUPS to the pollen tube’s tip. This mechanism activates the ROP2–RBOHH/J pathway via the autocrine signaling of RALF4/19, maintaining ROS generation and cell wall integrity (Feng et al. 2019).

Under conditions of high-salt and high-temperature stresses, *fer* and *llg1* mutants exhibited increased sensitivities, compared to wild-type *Arabidopsis thaliana*. Recent studies involving phase separation have shed light on this phenomenon. Specifically, RALF-pectin phase separation under stress conditions induces FER and LLG1 clustering, and endocytosis. This leads to both cognate and non-cognate receptor clustering and promiscuous endocytosis in a manner dependent on FER and LLG1, a crucial process for the recovery from stress-induced growth attenuation (Liu et al. 2024). The intricate RALFs–LRE/LLGs–CrRLK1Ls signaling modules reveal a novel peptide perception mechanism in plants. This involves the collaboration of GAPs with receptor kinase, forming unique heterocomplexes with CrRLK1Ls and LRE/LLGs, showcasing the diverse and sophisticated nature of plant peptide signaling pathways.

### RLCKs are essential peptide hormone signaling components

Receptor-like cytoplasmic kinases (RLCKs) are another type of RLKs, which lack an extracellular domain and are solely membrane-anchored or located in the cytoplasm (Liang and Zhou 2018). RLCKs have been found to play key roles in multiple RLK-mediated signaling pathways, such as brassinosteroid (BR) signaling, immune responses, reproductive process (Sreeramulu et al. 2013; Kong et al. 2016; Liao et al. 2016; Liang and Zhou 2018). Recently, a group of RLCKs, including PBS1-LIKE34/35/36 (PBL34/35/36), were identified to be essential for inhibiting protophloem differentiation in the root tip (Wang et al. 2022c). The *pbl34/35/36* triple mutant exhibited reduced sensitivity to CLE25/45 peptide treatment and could partially rescue the short-root phenotype of *brx* and *ops*. PBL34/35/36 can interact with BAM1/3 and be phosphorylated by BAM1. In summary, receptors BAM1 and BAM3 perceive extracellular peptides CLE25/26/45 to recruit and phosphorylate co-receptors CIKs. This signaling cascade is further transmitted to phosphorylate intracellular PBL34/35/36, which may in turn phosphorylate downstream signaling elements, ultimately regulating protophloem differentiation and maintaining proximal root meristem homeostasis (Wang et al. 2022c) (Fig. 3).

### Structural basis for peptide hormone perception by their receptors

Although extensive genetic, biochemical, and physiological data have demonstrated that peptide hormones are perceived usually by receptor–co-receptor RLK complexes, and these signals are transduced into the cell through phosphorylation, how the signaling cascades are initiated once the ligand perception occurs is still largely unknown. In the past decade, structural analyses of several RLK receptors involved in small peptide perception facilitated our understanding of this critical step (Chakraborty et al. 2019; Wang and Chai 2020). Some LRR-RLKs possess an island domain in their extracellular domain, which collaborates with the LRR core region to facilitate ligand binding. For instance, PSKR1 exhibits an external island domain that remains flexible in the absence of PSK ligand. Upon PSK perception, this island domain becomes more stable, facilitating the recruitment of co-receptor SERK to accomplish signal transduction (Matsubayashi et al. 2002, 2006; Wang et al. 2015b). Some LRR-RLKs lack an island structure in their extracellular domain but solely rely on direct binding between their LRR domain and the ligand. For example, when IDA binds to the receptor HAE, IDA extends along the inner surface of HAE extracellular LRRs. Remarkably, IDA possesses conserved amino acid residues arginine, histidine, and asparagine at its C-terminal, which interact with extracellular arginine and aspartate residues of HAE during ligand perception. Arginine and aspartate residues are commonly conserved in the extracellular domain among many other RLK receptors, suggesting a potential paradigm of ligand perception by RLKs (Butenko et al. 2003, 2014; Santiago et al. 2016). Additionally, hydroxyproline modification on central proline residues within IDA is essential for its efficient binding to HAE by promoting hydrogen bond formation with HAE (Santiago et al. 2016). TDIF/CLE41/44, is perceived by TDR/PXY and its co-receptor SERK to maintain vascular stem cells (Fisher and Turner 2007; Hirakawa et al. 2008; Etchells and Turner 2010; Zhang et al. 2016b). CLE41/44 bind to the inner surface of PXY LRR domain in the form of “Ω” and interact with GxY motif and DxSxN motif of PXY. These motifs are conserved in other CLE receptors, which suggests a possible perception pattern of these CLE peptides by their receptors. After perception, CLE41/44 stabilize the interactions between PXY and SERK by forming hydrogen bonds between CLE41/44 C-terminal Ser11 and SERK2 Thr56 and Val58 (Zhang et al. 2016a, b). RGF1 presents a fully extended conformation. Both terminals of RGF1 interact with two positive patches in the small peptide binding region of RGI, while the middle region of RGF1 interacts with negatively charged patches of RGI. Moreover, the RxR motif of RGI can interact with asparagine at the end of RGF1, which plays a role in recognizing RGF1 (Song et al. 2016). During sexual plant reproduction, pollen-specific receptor kinase 6 (PRK6) senses small peptide LURE1.2 to regulate ovule attraction of pollen tubes (Okuda et al. 2009; Takeuchi and Higashiyama 2012, 2016). A disulfide bond between Cys237 and Cys229 stabilizes the C-terminal loop of PRK6 LRR domain, which plays a role in the interaction with AtLURE1.2 (Zhang et al. 2017).

Structural analyses have also been performed for a few ligand–receptor pairs involved in immune responses. The terminal residues of flg22 can bind to the concavity of FLS2 LRR domain. Both flg22 terminals can be recognized by conserved and non-conserved sites of extracellular FLS2. The co-receptor BAK1 can recognize the C-terminal of FLS2 bound by flg22 (Gómez-Gómez and Boller 2000; Asai et al. 2002; Chinchilla et al. 2007; Sun et al. 2013). PEP1 RECEPTOR 1 (PEPR1) and PEPR2 sense PEPs to regulate DAMP-induced immune responses (Huffaker et al. 2006; Yamaguchi et al. 2006, 2010; Krol et al. 2010). AtPep1 extends along the inner surface of the LRR domain of PEPR1 extracellular superhelix and binds to PEPR1 through its conserved C-terminal. AtPep1 interacts with PEPR1 LRR through the terminal asparagine (Tang et al. 2015). At present, there are few structural studies on how non-LRR receptor kinases sense small peptide ligands. For example, chitin regulates immune processes by interacting with extracellular lysine motif (LysM) of CHITIN ELICITOR RECEPTOR KINASE1 (CERK1) (Miya et al. 2007; Willmann et al. 2011). Only the extracellular LysM2 structure of CERK1 binds to chitin, and chito-oligomers are often anchored to the shallow groove formed by LysM2 (Liu et al. 2012). RALF23 regulates immune responses by being sensed by the FER-LRE/LLG1 complex(Pearce et al. 2001b; Li et al. 2015; Stegmann et al. 2017). LLG1/2 can directly recognize the conserved N-terminal region of RALF23 and bind to FER to form a complex. The N-terminal sequence of RLAF23 is sufficient to induce the extracellular region of FER to interact with LLG1/2/3, and the C-terminal of RALF23 is able to consolidate the interaction between FER and LLG (Xiao et al. 2019).

Up to date, structural results were reported for only a few RLK receptors of peptide hormones. Especially, ligands of numerous RLKs have not been identified yet. It can be expected that more interaction patterns between peptide hormones and RLK receptors will be revealed in the future. At the same time, structural basis for co-receptor RLK functions is far from enough for further understanding their critical roles in peptide hormone signaling pathways, especially how co-receptors affect recognitions between peptide hormones and receptors. A huge challenge is to study structure transformation of a receptor complex forming with intact receptor and co-receptor upon peptide hormones perception in planta.

### The antagonistic roles of peptides fine-tuning plant development

**Fig. 4:**
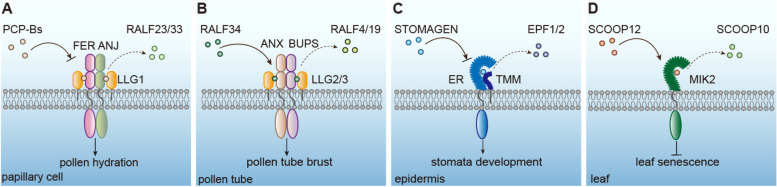
Fine-tuning of plant development through antagonistic peptides. **A** model of pollen PCP-B peptides repression of stigma RALF23/33 peptide during pollen-stigma interaction. Before pollination, RALF23/33 induces ROS production in the stigmatic papillary cells through a FER/ANJ-controlled signaling pathway. Upon pollination, the competitive binding of pollen PCP-Bs to the stigma ANJ–FER receptor complex displaces stigmatic RALF23/33, repressing ROS production and thus stimulating pollen hydration. **B** Autocrine peptides RALF4/19 in the pollen tube activate the BUPS1/2–ANX1/2–LLG2/3 receptor complexes, ensuring the maintenance of cell wall integrity and promoting pollen tube growth within the transmitting tract. Upon reaching the micropyle, the ovule-secreted RALF34 competes with RALF4/19 for binding to BUPs/ANXs receptors, leading to pollen tube bursting and successful fertilization. **C** the competitive binding of STOMAGEN and EPF2 to the ER receptor kinase, along with its coreceptor TMM, finely tunes the initiation of stomatal development. The ER receptor kinase perceives the EPF2 peptide, leading to the inhibition of stomatal formation. Concurrently, STOMAGEN competes with EPF1/2 for the binding sites on the ER–TMM complex, positively regulating stomatal development. **D** the antagonistic regulation of leaf senescence by SCOOP10 and SCOOP12 peptides occurs via the MIK2 receptor-like kinase. MIK2 functions as a negative regulator of leaf senescence, with SCOOP10 promoting senescence by inhibiting MIK2 phosphorylation during the early stages. Conversely, SCOOP12 competes with SCOOP10 for binding to the MIK2 receptor, activating its phosphorylation during later stages of senescence, thereby suppressing senescence

In the *A. thaliana* genome, more than 1000 genes encoding putative small, secreted signaling peptides have been identified, which may regulate diverse aspects of plant growth and development (Lease and Walker 2006). Several studies have revealed that the peptides, belonging to the same or different families, can act antagonistically in fine-tuning specific biological processes (Lee and De Smet 2016). In tomato (*L. esculentum*), prior to pollen germination, the pollen receptor kinase 2 (LePRK2) interacts with a pollen-specific CRP, LATE ANTHER TOMATO52 (LAT52), to regulate germination (Tang et al. 2002; Johnson and Preuss 2003); Post germination, LePRK2 binds to STIGMA-SPECIFIC PROTEIN1 (LeSTIG1), a CRP from the stigmatic secretory zone, where exogenous LeSTIG1 can disrupt the LAT52-LePRK2 interaction in mature pollen, suggesting that LeSTIG1 might replace LAT52 upon pollen germination on the stigma (Tang et al. 2015). In *Arabidopsis thaliana*, antagonistic peptides from different families regulate the recognition process during pollination by competing for the same receptor. Stigmatic RALF23/33 peptides bind to FER and ANJ receptors, triggering a ROS-producing pathway that acts as a gatekeeper before pollination (Song et al. 2022). Compatible pollen grains carrying pollen coat protein B-class (PCP-B) peptides then compete with RALF23/33 for binding to FER-ANJ, reducing ROS levels and initiating pollen hydration and germination (Liu et al. 2021a) (Fig. 4). Within the same peptide family, the EPFs demonstrate how homologous peptides can have opposing effects. EPF1 and EPF2 negatively regulate stomatal development by interacting with specific receptors (ER, ERL1, and ERL2), whereas their homolog, STOMAGEN/EPFL9, acts antagonistically to promote stomatal development by binding to the ER receptor and blocking EPF2’s action (Lee et al. 2012, 2015; Yu et al. 2023a) (Fig. 4). It has also been identified that compatible pollen-derived RALFs (pRALFs) displace stigmatic RALFs to facilitate pollen tube entry, a mechanism that has implications for overcoming reproductive barriers in distant hybridization breeding (Lan et al. 2023). Homologous antagonistic RALFs also regulate pollen tube integrity, with RALF4/19 maintaining tube integrity via the ANXs/BUPSs–LLG2/3–RBOHD pathway, and their action being opposed by female-derived RALF34 to induce pollen tube rupture upon contact with the female gametophyte (Ge et al. 2017; Feng et al. 2019) (Fig. 4). Recently, two SCOOP peptide family members, SCOOP10 and SCOOP12, have been identified as antagonistic regulatory factors that fine-tune the senescence process in *Arabidopsis thaliana* leaves via the MIK2 receptor, with SCOOP12 exhibiting a stronger competition for binding to the MIK2 receptor, leading to a dramatic inhibition of SCOOP10-induced leaf senescence (Zhang et al. 2024c) (Fig. 4). Meanwhile, the CLE signaling pathway mediated by BAM in the root meristem has also been reported to exhibit antagonistic regulatory activity, where BAM3-meidated CLE45 signaling counteracts BAM1/2-mediated CLE11/12/13 signaling specifically in the phloem initials, but not in the ground tissue (Zhang et al. 2024b).

Other than the natural antagonistic peptides exist in plants, Song et al. (2013) developed antagonistic peptides through a glycine-to-threonine substitution in the conserved CLE motif (Song et al. 2013). Intriguingly, the conserved glycine residue was also found in several other types of peptide hormones, making it possible to manipulate these peptides to create antagonistic peptides. The competitive regulatory mechanisms demonstrated by these examples serve as potent tools for controlling receptor activities and specific plant cell developmental processes. Further studies in structural and cell biology are essential to address the conservations and specificities of these antagonistic interactions.

## Conclusions and perspective

Since the first peptide hormone, systemin, was discovered in plants in 1991 (Pearce et al. 1991), many peptide and putative peptide hormones have been discovered in plants. The knowledge accumulated so far suggest that peptide hormones play critical roles in short-distance intercellular communications, to coordinate cellular behavior such as stem cell homeostasis, cell divisions, tissue differentiations and programmed cell death in multi-cellular flowering plants, in response to intrinsic and extrinsic cues. These peptides also play important roles in systematic and long-distance signal transductions, to communicate between roots and shoots, between different organs, between plant and pathogen, and between pollen grain and ovule. As read from this article, through actions and collaborations of many groups in the world, substantial progresses have been made in these areas. However, many challenges remain to be tackled.

1. Although many putative small peptides have been identified in different ways in plants, only a small fraction of them can really be assigned as peptide hormones, with clearly defined roles both in vivo and in vitro, expression patterns, mature forms, and receptors (or receptor complexes) known.
2. Most of these peptides identified so far are encoded by multiple genes, and mutations in one or a few of them often showed no detectable phenotype, which bring out the questions that how the functions of these redundant genes are evolved in nature, and what is the role of each individual gene, and how to decipher their functions?
3. Since most peptide hormones are expected to be present in plants in very low concentrations, new technologies are needed to identify and analyze their endogenous forms.
4. Almost all peptide hormones are post-translational secreted, cleaved, and/or modified in different forms, new tools are needed to understand how these processing are executed, and if such modifications are for peptide stabilities, peptide-receptor interactions, or degradations.
5. Multiple receptor kinases are identified for sensing one or a family of peptides. How the actions of these receptor kinases are coordinated to sense one or multiple peptides, and whether they form a complex for sensing these peptides, or the complexes are formed in response to peptide hormones.
6. We know very little about the downstream signal transduction pathways that relay the peptide signal from receptor kinases to cellular responses and gene expressions.
7. The advent of sequencing-based technologies has greatly enhanced our understanding of the non-canonical peptides derived from previously recognized non-coding regions. Nevertheless, despite their abundance in plants, only a limited number of these peptides have been thoroughly studied for their functional roles and the mechanisms underlying the translation of these unannotated regions remain largely unknown.
8. The differences between peptide signals and traditional small-molecule phytohormones remain poorly understood. Furthermore, the interaction/crosstalk between peptide hormones and conventional phytohormones have yet to be thoroughly investigated.

## Data Availability

Not applicable.

## Abbreviations

ACR4: Arabidopsis crinkly4
AHA2: H+-adenosine triphosphatase 2
ANJ: Anjea
ANX1/2: Anxur 1/2
AON: Auto-regulation of nodulation
ArafTs: Arabinofuranosyltranferases
BAMs: Barely any meristems
BR: Brassinosteroid
BUPS1/2: Buddha’s paper seal1/2
CAPE: Cap-derived peptide
CEI: Cortex/endodermis initial
CEPs: C-terminally encoded peptides
CERK1: Chitin elicitor receptor kinase1
cGMP: Cyclic guanosine monophosphate
CIF: Casparian strip integrity factor
CIKs: Clavata3 insensitive receptor kinases
CLERK: Cle-resistant receptor kinase
CLEs: Clavata3/embryo surrounding region-related peptides
CLV3: Clavata3
CPS: Conventional protein secretion
CRA2: Compact root architecture 2
CRN: Clv2-coryne
CRPs: Cysteine-rich proteins
CrRLK1L: Catharanthus roseus receptor-like kinase 1-like
Cys-rich: Cysteine richness
DAMPs: Damage-associated molecular patterns
DEFLs: Defensin-likes
DVL1: Devil1
ECL: Egg cell 1-like
EFR: Ef-tu receptor
ENOD40: Early nodulin 40
EPFL: Epf-like
EPFs: Epidermal patterning factors
ER: Endoplasmic reticulum
ERF115: Ethylene response factor 115
ERL1: Erecta-like 1
ES: Embryo sac
ES4: Embryo sac4
ESF1: Embryo surrounding factor1
EXPO: Excyst-positive organelles
FCP1: Fon2-like cle protein1
flg22: Flagellin 22
FLS2: Flagellin sensitive 2
FON2: Floral organ number2
FRE: Feronia
*GAD1*: Grain length and awn development1
GAPs: Gpi-anchored proteins
GASA: Gibberellic acid stimulated in Arabidopsis
*GL2*: Glabra 2
Gly: Glycine
GPI: Glycosylphosphatidylinositol
GRI: Grim reaper peptide
GRP: Glycine-rich protein
GSO1/2: Gassho1/2
HAE: Haesa
HAR1: Hypernodulaton and aberrant root 1
HERK1: Hercules receptor kinase 1
HLP: Hevein-like peptide
HSL2: Haesa-like 2
HypSys: Hydroxyproline-rich systemin
IDA: Inflorescence deficient in abscission
IDL: Ida-like
KLP: Knottin-like peptide
KOD: Kiss of death
LAT52: Late anther tomato52
LePRK2: Pollen receptor kinase 2
LLGs: Lorelei-like-gpi-anchoredproteins
LRE: Lorelei
LRR: Leucine-rich repeat
LTPs: Lipid transfer proteins
Lys: Lysine
LysM: Lys motif
MAPK: Mitogen-activated protein kinase
MCs: Metacaspases
miPEP: miRNA-encoded peptide
MLD: Malectin-like domains
MPK6: Map kinase 6
NCR: Nuclear transcription factor y subunit a
NF-YA: Non-specific lipid transfer proteins
nsLTPs: Non-specific lipid transfer proteins
ORFs: Open reading frames
OSIP: Oxidative stress-induced peptide
P2: Position 2
P4Hs: Prolyl-4-hydroxylases
PBL: Pbs1-like
PCP-B: Pollen coat protein b-class
PDFs: Plant defensins
PEPs: Plant elicitor peptides
PHR1: Phosphate starvation response 1
Pi: Phosphate
PIP: Pamp-induced secreted peptide
PIPLs: Pip-likes
PLS: Polaris
PLT: Plethora
PNP: Plant natriuretic peptide
pRALF: Pollen-expressed ralf
PRK6: Pollen-specific receptor kinase 6
Pro: Proline
proIDA: Ida precursor
PS: Prosystemin
PSKR1: Psk receptor-1
PSKs: Phytosulfokines
PSY: Plant peptides containing sulfated tyrosine
PTI: Pamp-induced immunity
PTMs: Post-translational modifications
PVY: Potato virus y
PXL1: Pxy-like1
PXY: Phloem intercalated with xylem
RALFs: Rapid alkalinization factors
RAM: Root apical meristem
REF1: Regeneration factor1
RGFR: Rgf receptor
RITF1: Rgf1-inducible transcription factor 1
RLCKs: Receptor-like cytoplasmic kinases
RLKs: Receptor-like protein kinases
ROS: Reactive oxygen species
ROT4: Rotundifolia4
RPK2: Receptor-like kinase 2
SAM: Shoot apical meristem
SBTs: Subtilisin-like serine proteases
SCOOPs: Ser-rich endogenous peptides
*SCR*: Scarecrow
SCR/SP11: S-locus cysteine-rich protein/s-locus protein11
SERK: Somatic embryogenesis receptor-like kinase
*SHR*: Short root
sRALF: Stigma-expressed ralf
SSPs: Small secreted peptides
STIG1: Stigma-specific protein1
SUBPEP: Subtilisin-embedded plant elicitor peptide
SUNN: Super numeric nodules
SYS: Systemins
TDIF: Tracheary element differentiation inhibitory factor
TDR: Tdif receptor
TGN: Trans-golgi network
THL: Thionin-like
TOR: Target of rapamycin
TPST: Tyrosylprotein sulfotransferase
TWS1: Twisted seed1
Tyr: Tyrosine
UPS: Unconventional protein secretion
*WOX5*: Wuschel-related homeobox5
WUS: Wuschel
ZIP1: Zea mays immune signaling peptide 1
2SA: 2s albumin
